# Cost–utility analysis of adjunctive psychosocial therapies in bipolar disorder

**DOI:** 10.1192/bjo.2025.10068

**Published:** 2025-07-21

**Authors:** Mary Lou Chatterton, Yong Yi Lee, Long Khanh-Dao Le, Melanie Nichols, Michael Berk, Cathrine Mihalopoulos

**Affiliations:** School of Public Health and Preventive Medicine, Monash University, Melbourne, Australia; Institute for Health Transformation, Deakin University, Geelong, Australia; School of Public Health, The University of Queensland, Herston, Australia; Queensland Centre for Mental Health Research, Brisbane, Australia; Institute for Mental and Physical Health and Clinical Translation, School of Medicine, Deakin University, Geelong, Australia

**Keywords:** Bipolar disorder, cost-utility analysis, psychosocial therapy, mania, depression

## Abstract

**Background:**

There are few economic evaluations of adjunctive psychosocial therapies for bipolar disorder.

**Aims:**

Estimate the cost–utility of in-person psychosocial therapies for adults with bipolar disorder added to treatment as usual (TAU), from an Australian Government perspective.

**Method:**

We developed an economic model, estimating costs in 2021 Australian dollars (A$) and outcomes using quality-adjusted life-years (QALYs) gained and disability-adjusted life-years (DALYs) averted. The model compared psychoeducation, brief psychoeducation, carer psychoeducation, cognitive–behavioural therapy (CBT) and family therapy when added to TAU (i.e. pharmacotherapy) over a year for adults (18–65 years) with bipolar disorder. The relative risk of relapse was sourced from two network meta-analyses and applied to the depressive phase in the base case. Probabilistic sensitivity analysis and one-way sensitivity analyses were conducted, assessing robustness of results.

**Results:**

Carer psychoeducation was preferred in the base case when the willingness-to-pay (WTP) threshold is below A$1000 per QALY gained and A$1500 per DALY averted. Brief psychoeducation was preferred when WTP is between A$1000 and A$300 000 per QALY gained and A$1500 and A$450 000 per DALY averted. Family therapy was only preferred at WTP thresholds above A$300 000 per QALY gained or A$450 000 per DALY averted. In sensitivity analyses, brief psychoeducation was the preferred therapy. Psychoeducation and CBT were dominated (more costly and less effective) in base-case and sensitivity analyses.

**Conclusions:**

Carer and brief psychoeducation were found to be the most cost-effective psychosocial therapies, supporting use as adjunctive treatments for adults with bipolar disorder and their families in Australia.

Bipolar disorder is a complex mental health condition associated with significant disability that leads to increased healthcare costs and adverse impacts on society.^1–3^ Pharmacotherapy is the recommended first-line maintenance treatment for bipolar disorder,^4,5^ but there have been few novel medications for bipolar disorder in the past decade. Medication adherence is often problematic,^6^ relapse rates are high and full remission evades many.^7^ This has led to the increasing importance of adjunctive therapies used alongside pharmacotherapy for the treatment of bipolar disorder.

Several psychosocial therapies have been evaluated as adjuncts to pharmacotherapy for bipolar disorder. Current clinical guidelines recommend psychoeducation as a first-line adjunctive treatment during the maintenance phase.^5^ Cognitive–behavioural therapy (CBT) and family-focused therapy are recommended as second-line adjunctive treatments for both maintenance and depressive phases.^5^ Interpersonal and social rhythm therapy and peer support are recommended as third-line adjunctive treatments, whereas mindfulness-based cognitive therapy and online interventions are not supported by clinical guidelines because of insufficient evidence.^5^

There have been four previous economic evaluations of adjunctive psychosocial therapies for bipolar disorder.^8–11^ A within-trial cost-effectiveness analysis of cognitive therapy compared with standard care in 103 participants diagnosed with type 1 bipolar disorder, found that those receiving cognitive therapy spent significantly fewer days with bipolar episodes and had lower healthcare costs over 30-month follow-up.^8^ However, this analysis is limited by the lack of value-for-money connotations associated with bipolar-free days as an outcome measure. A model-based cost-effectiveness study of lithium and valproate with and without psychosocial care across 14 global regions, found community-based delivery of lithium and psychosocial care combined was cost-effective relative to valproate and psychosocial care, either lithium or valproate alone, or the same therapies delivered through a hospital-based service model.^9^ An additional model-based economic evaluation of interventions to address five neuropsychiatric conditions, including bipolar disorder, in Sub-Saharan Africa and South-East Asia found lithium and psychosocial care delivered through a community model was cost-effective compared with valproate and psychosocial care delivered through a community model or either medication and psychosocial care provided through a hospital-based model of care.^10^ Both modelled economic evaluations^9,10^ lacked clarity regarding the health professionals delivering the psychosocial therapy and whether the sessions were provided individually or in groups. An additional within-trial cost–utility analysis found that structured group psychoeducation was not cost-effective compared with group peer support.^11^ This study also used an economic model to evaluate the group psychoeducation intervention compared with treatment as usual (TAU). It found that psychoeducation had an average incremental cost per quality-adjusted life-year (QALY) gained below the £30 000 willingness-to-pay (WTP) threshold and may even be cost-saving. However, the uncertainty around the incremental cost-effectiveness ratios (ICERs) led to probabilities of ≤54% that psychoeducation would be cost-effective compared with TAU.

There is currently limited evidence around which adjunctive psychosocial therapies for bipolar disorder are among the most cost-effective options in the Australian setting. The aim of this study is to assess the comparative cost–utility of five different, mutually exclusive psychosocial therapies as adjuncts to TAU (i.e. pharmacotherapy) for adults with bipolar disorder, when adopting an Australian Government health payer perspective.

## Method

### Analytic approach

The current analysis used a standardised economic evaluation framework, based on the technical methods of the broader Assessing Cost-Effectiveness (ACE) approach to priority setting, previously used to model healthcare interventions and inform healthcare service delivery in Australia.^12^ A decision-analytic model was developed to estimate costs and health outcomes associated with managing bipolar disorder among Australian adults aged 18–65 years.^13^

A cost–utility framework was used, in which outcomes were expressed as QALYs gained, as well as disability-adjusted life-years (DALYs) averted.^14^ The QALY is a composite health outcome jointly measuring mortality and morbidity impacts. It is widely used in economic evaluations for health technology assessment agencies such as the UK National Institute for Health and Care Excellence (NICE) and the Australian Pharmaceutical Benefits Advisory Committee (PBAC). The DALY is an alternative composite health outcome also jointly measuring mortality (years of life lost) and morbidity impacts (years lived with disability).^15^ The DALY has been used in previous economic evaluations to measure population health loss, assess incremental health outcomes and optimise the mix of healthcare services provided.^9^

The perspective adopted is from the Australian Government as a third-party payer of healthcare services. The evaluation excluded costs to the private sector, non-government organisations, out-of-pocket costs to patients, and non-health sectors (e.g. welfare, housing).

There is no explicit WTP threshold adopted in Australia for decision-making.^12^ Previous Australian economic evaluation studies have used a WTP threshold of $50 000 Australian dollars (A$) per QALY gained (or per DALY averted) as a rule of thumb.^12^ We adopted a range of WTP thresholds used by the Australian Productivity Commission Inquiry into Mental Health. These thresholds were defined as: A$33 000 per QALY gained (very cost-effective); <A$64 000 per QALY gained (cost-effective); and <A$96 000 per QALY gained (marginally cost-effective).^16^ The same WTP thresholds were used to compare results involving DALYs averted given the previous adoption of a common threshold for both outcome measures.^12^

### Intervention descriptions and effectiveness

The psychosocial therapies modelled in this study included those identified as effective in reducing the risk of manic or depressive relapse/recurrence compared with TAU (i.e. pharmacotherapy) across two network meta-analyses (NMAs).^17,18^ Both NMAs collected data on manic or depressive relapse from randomised controlled trials of psychosocial therapies for the treatment of bipolar disorder (including types 1, 2 and cyclothymia) in the acute or maintenance phase among adults (≥18 years) receiving regular mood-stabilising medication at intake. Variations in the classification of interventions, analysis methods and included studies led to differences in results. The NMA by Chatterton et al,^17^ which estimated the relative risk of relapse using an inverse variance heterogeneity model, found that only carer-focused interventions (hereafter referred to as carer psychoeducation) were effective in preventing relapse relative to TAU.^17^ Carer psychoeducation is provided to carers or family members of people with bipolar disorder without the person with bipolar disorder present. The NMA by Miklowitz et al,^18^ which estimated odds ratios using a random-effects model, found that four psychosocial therapies including family or conjoint therapy (hereafter referred to as family therapy), psychoeducation, brief psychoeducation and CBT were all effective in reducing the recurrence of depression or mania compared with TAU.^18^ We utilised the data from Miklowitz et al^18^ to estimate the relative risk of relapse associated with each effective psychosocial therapy in MetaXL (version 5.1 for Windows, EpiGear International Pty, Sunrise Beach, Australia; https://www.epigear.com/index_files/metaxl.html). We first estimated odds ratios through NMA by using a random-effects model to replicate results from Miklowitz et al (Supplementary Appendix 1, Table 1 available at https://doi.org/10.1192/bjo.2025.10068). Relative risk was estimated by using a random-effects model (Supplementary Appendix 1, Table 2), and finally, using the inverse variance heterogeneity model (Supplementary Appendix 1, Table 3). The relative risk of relapse for each intervention estimated with the inverse variance heterogeneity model was used in the base case of the economic evaluation. This method was chosen as it can produce a less biased estimator and a more conservative confidence interval than the random-effects model.^19^ CBT had a non-significant relative risk when using the inverse variance heterogeneity model, but was included in the economic evaluation because of the significant finding by Miklowitz et al.^18^ The final list of included interventions with estimated relative risk of relapse are provided in Table 1.

**Table 1:** Intervention descriptions and effect sizes

Intervention	Description	Relative risk (95% CI)	Source
Psychoeducation	Defined as six or more sessions (either individual- or group-based) where patients (or family members) are provided with information on: illness features; importance of treatment adherence; early detection of prodromal signs of recurrence; management of mood symptoms or comorbid conditions; and lifestyle regularity	0.80 (0.67–0.95)	Adapted from Miklowitz et al, 2021^18^
Brief psychoeducation	Defined as fewer than three psychoeducation sessions (to groups, families or individuals) and was designated as a control treatment in some trials	0.48 (0.27–0.85)	Adapted from Miklowitz et al, 2021^18^
Carer psychoeducation	Sessions where only carers or family members of a person with bipolar disorder are provided information on the symptoms, nature, type and length of bipolar disorder treatment. The outcomes were assessed among the people with bipolar disorder	0.61 (0.44–0.86)	Chatterton et al, 2017^17^
Cognitive–behavioural therapy	Aims to modify maladaptive thoughts through cognitive restructuring. It also includes psychotherapeutic components such as mindfulness and acceptance and commitment therapy	0.84 (0.64–1.11)	Adapted from Miklowitz et al, 2021^18^
Family therapy	Involves the joint delivery of intervention sessions to patients and their caregivers (i.e. parents, spouse or extended relatives). The sessions were delivered to a single-family or multi-family group and included a mixture of psychoeducation, communication and problem-solving skills training	0.43 (0.30–0.62)	Adapted from Miklowitz et al, 2021^18^

### Target population

The target population comprised adults in Australia, aged 18–65 years with bipolar disorder, seeking treatment through public or private community-based care. It was estimated by taking the 2019 Australian population (by 1-year age and gender),^20^ applying the Australian prevalence of bipolar disorder (types 1 and 2) from the Global Burden of Disease Study 2019 (GBD),^21^ and limiting it to the estimated proportion of people with bipolar disorder seeking medical care (67.7%).^22^

### Modelling health outcomes

An economic model was designed in Microsoft Excel to simulate health state transitions experienced by population cohorts with bipolar disorder (i.e. the target population) with and without the delivery of each adjunctive psychosocial therapy over 1 year. The number of individuals with bipolar disorder was adjusted for the annual all-cause mortality and additional bipolar disorder-related mortality (Table 2).^23,24^ The health states included a residual state (stable), mania and depression. The time spent in each health state when receiving TAU for bipolar disorder over the course of 1 year was taken from a meta-analysis.^25^ This resulted in TAU encompassing 3.2 months (27%) in a depressed state, 2.8 months (23%) in a manic state and 6 months (50%) in a residual state. The time spent in each health state when receiving a specific psychosocial therapy was modified based on the relative risk of relapse. The risk of relapse from the two NMAs was not specific to mania or depression. As such, the base-case analysis applied the relative risk of relapse to the depressive phase, given that more time is spent in the depressive phase.

**Table 2:** Input parameters and uncertainty ranges

Parameter	Value and uncertainty range	Distribution	Source
Population	18–65 years	Not applicable	Australian Bureau of Statistics, 2020^20^
All-cause mortality	Age- and gender-specific rates	Fixed	Australian Bureau of Statistics, 2021^23^
Bipolar disorder-specific mortality	1.64 (males), 1.59 (females)	Fixed	Angst et al, 2002^24^
Prevalence of bipolar disorders	Age- and gender-specific prevalence	Fixed	IHME, 2023^21^
Relative risk of relapse after psychoeducation	0.80 (0.67–0.95)	PERT	Adapted from Miklowitz et al, 2021^18^
Relative risk of relapse after brief psychoeducation	0.48 (0.27–0.85)	PERT	Adapted from Miklowitz et al, 2021^18^
Relative risk of relapse after carer psychoeducation	0.61 (0.44–0.86)	PERT	Chatterton et al, 2017^17^
Relative risk of relapse after cognitive–behavioural therapy	0.84 (0.64–1.11)	PERT	Adapted from Miklowitz et al, 2021^18^
Relative risk of relapse after family therapy	0.43 (0.30–0.62)	PERT	Adapted from Miklowitz et al, 2021^18^
Disability weight bipolar disorder residual state	0.032 (0.018–0.051)	Beta	IHME, 2020^30^
Disability weight bipolar disorder manic episode	0.429 (0.341–0.646)	Beta	IHME, 2020^30^
Disability weight major depressive disorder moderate episode	0.396 (0.267–0.531)	Beta	IHME, 2020^30^
Utility weight bipolar disorder residual state	0.8 (0.755–0.845)	Beta	Revicki et al, 2005^29^
Utility weight bipolar disorder manic episode	0.54 (0.42–0.65)	Beta	Revicki et al, 2005^29^
Utility weight bipolar disorder depressive episode	0.29 (0.16–0.42)	Beta	Revicki et al, 2005^29^

Disability weights quantify social preferences for different health states and are used to calculate disability-adjusted life years (DALYs). They range from: 0 (denoting full health) to 1 (denoting death). Utility weights quantify social preferences for different health states and are used to calculate quality-adjusted life years (QALYs). They range from: 0 (denoting death) to 1 (denoting full health).

### Cost analysis

Healthcare resources were costed according to Australian guidelines.^26^ The number and length of psychosocial therapy sessions and the type of healthcare provider delivering sessions were extracted from trials included in the two NMAs (Supplementary Appendix 1, Table 4). Several assumptions were made to estimate the cost of intervention delivery, given heterogeneity in intervention design observed across trials. All intervention costing was based on the weighted average number of sessions from included trials (shown in Supplementary Appendix 1, Table 4). Carer psychoeducation was costed as eight group sessions only involving carers, with the assumption that every person with bipolar disorder would have one carer or family member participate in sessions. Family therapy costs were calculated for 18 individual family sessions delivered by a clinical psychologist, based on results from seven trials. Four of nine primary psychoeducation studies utilised group sessions with experienced psychiatric staff. We costed these as 14 sessions with a clinical psychologist, with 50% in a group format and 50% as individual sessions. The CBT intervention costing comprised 20 individual sessions with a clinical psychologist, given that nine of ten studies utilised individual delivery. Brief psychoeducation sessions were costed as three individual sessions with a clinical psychologist (Supplementary Appendix 1, Table 5).

The average cost to the Australian Government for individual- and group-based psychological therapy services delivered by a clinical psychologist was estimated through government reimbursement records for the period from July 2021 to June 2022. These unit costs were combined with the number of intervention sessions to estimate the total cost of intervention delivery (Supplementary Appendix 1, Table 5).

Resource use for the standard care of people with bipolar disorder who relapse to manic and depressed health states was estimated by using descriptions of optimal treatment obtained through a panel of experts (Supplementary Appendix 1, Table 6).^27^ This was chosen since there are no resource use or cost estimates by bipolar disorder phase available in Australia. The panel estimated the proportions likely to receive specific treatments and the number of services by health state. Total costs were calculated as a weighted average based on population proportions estimated to use each service. Unit costs for healthcare consultations with general practitioners and private psychiatrists were sourced from the Australian Medicare Benefits Schedule (MBS). An average cost for a community mental health visit was estimated based on the total cost of community mental health services divided by the number of community health services provided in 2019/20. In-patient admissions were costed using the average cost for a public hospital admission under Australian refined diagnosis-related group (AR-DRG) U63A (Major Affective Disorders, Major Complexity) sourced from the 2018/19 National Hospital Cost Data Collection. Unit costs were inflated to 2021 prices with the health price index.^28^ The use and cost of clinical psychologists by 10% of the population in the acute depressive phase was removed from the costing to avoid double counting.

### Cost–utility analyses

Outcomes were expressed as QALYs. These were calculated by using utility weights measuring health gains on a scale between 0 (denoting death) and 1 (denoting full health). Only one study from the USA estimated utility weights for each bipolar disorder state (i.e. mania, depression and residual), using the standard gamble technique.^29^ Utility weights were 0.80 (95% CI 0.76–0.85) residual state, 0.54 (95% CI 0.42–0.65) manic state and 0.29 (95% CI 0.16–0.42) depressive state.

Outcomes were also expressed as DALYs by using disability weights obtained from the GBD.^30^ Disability weights measure health loss on a scale between 0 (denoting perfect health) and 1 (denoting death). The disability weights for the residual state (0.032; 95% CI 0.018–0.051) and manic state (0.492; 95% CI 0.341–0.646) were specific to bipolar disorder. The GBD did not have a specific disability weight for bipolar-related depression. As such, a disability weight for moderate major depressive disorder (0.396; 95% CI 0.267–0.531) was used.^30^

Total outcomes (expressed as QALYs/DALYs) and total costs were estimated for each of the five adjunctive psychosocial therapies, when compared with TAU. A cost-effectiveness frontier (aka efficiency frontier) was generated to evaluate the comparative cost-effectiveness between the five mutually exclusive therapies. This was done by first ranking each therapy option in ascending order of total costs, followed by ascending order of total outcomes. Therapies that cost more but produced lower outcomes than the next less-costly therapy were dominated and thus excluded from the frontier. The cost-utility of each adjacent therapy on the cost-effectiveness frontier was estimated as the difference in total costs between a given therapy and the next less-costly therapy, divided by the corresponding difference in QALYs gained (or DALYs averted). Therapies excluded from the frontier were assigned a ‘dominated’ ICER.

### Uncertainty and sensitivity analyses

Probabilistic sensitivity analyses were undertaken with Monte Carlo simulation to evaluate the impact of sampling uncertainty around the input parameters on cost–utility results. Input parameters with uncertainty are shown in Table 2. Ersatz software (version 1.35 for Windows, EpiGear International, Sunrise Beach, Australia; https://www.epigear.com/index_files/ersatz.html) was used to generate 5000 simulations and calculate 95% uncertainty intervals (UI) for total costs, QALYs gained, DALYs averted and ICERs.

The uncertainty around estimated mean ICERs was visually depicted by plotting outcomes and costs produced by each simulation on a cost-effectiveness plane.^31^ The resulting cost-effectiveness frontier was also depicted on the cost-effectiveness plane, with the origin depicting TAU. Simulations were also used to construct cost-effectiveness acceptability curves indicating the proportion of simulations where an intervention has the greatest net monetary benefit across a range of WTP thresholds.^32^ Net monetary benefit was calculated as the WTP threshold multiplied by the outcome (QALYs gained or DALYs averted) minus the cost.^32^ Cost-effectiveness acceptability frontiers were also constructed to show which psychosocial therapy had the maximum net monetary benefit across a range of WTP thresholds.^13^

Additional one-way sensitivity analyses determined the effect of modifying key parameters on results. This included applying the relative risk of relapse to the manic phase, using a unit cost for psychologists delivering the interventions (Supplementary Appendix 1, Table 7), using the relative risk of relapse for CBT estimated using a random-effects NMA and extending the benefits of CBT to 5 years. The rationale for using a psychologist unit cost was to provide policy makers additional information to assist in reimbursement decisions.

Since the time horizon was 1 year, costs were not discounted. The exception was for the sensitivity analysis extending the time horizon for CBT, which applied a 3% discount rate to both costs and outcomes incurred beyond year 1. This rate was chosen to align with previous ACE studies.^33^

### Ethics statement

The authors assert that all procedures contributing to this work utilise publicly available data and therefore complies with ethical standards of the National Statement on Ethical Conduct in Human Research (2007) and the Helsinki Declaration of 1975, as revised in 2013.

## Results

### Base-case analyses

Intervention effects were modelled for 190 155 Australian adults with bipolar disorder estimated to be seeking treatment in 2019. In the base-case analysis, carer psychoeducation had the lowest total cost (mean A$18.2 million, 95% UI A$1.9–36.0) and average cost per QALY gained (A$904, 95% UI A$117–$1512), shown in Table 3. Brief psychoeducation had an incremental cost per QALY gained of A$949 (95% UI Dominant to A$104 121) compared with carer psychoeducation. Family therapy had an incremental cost per QALY gained of A$285 856 (95% UI A$81 335 to Dominated) compared with brief psychoeducation. Psychoeducation was more costly and estimated to deliver fewer QALYs (mean 10 290; 95% UI 7924–12 669) than brief psychoeducation. Therefore, psychoeducation was dominated by brief psychoeducation. CBT was more costly than family therapy and had the lowest QALYs gained (mean 8241, 95% UI 4289–11 842), leading to CBT being dominated by family therapy.

**Table 3:** Results of base-case analyses for the outcome of quality-adjusted life-years (QALYs) gained

Intervention	Total costs (A$ 2021, millions)	Total QALYs	Incremental costs (A$ 2021)	Incremental QALYs	Incremental cost per QALY gained (A$ 2021)
	Mean (95% UI)	Mean (95% UI)	Mean (95% UI)	Mean (95% UI)	Mean (95% UI)
Carer psychoeducation, depression effect	18.2 (1.9–36.0)	20 108 (16 311–23 787)	–	–	–
Brief psychoeducation, depression effect	24.5 (2.6–48.5)	26 798 (21 481–31 733)	6.35 (−21.0 to 35.9)	6689 (288–12 993)	949 (Dominant^a^–104 121)
Family therapy, depression effect	776.3 (733.3–819.9)	29 428 (26 006–32 869)	751.8 (700.5–801.3)	2630 (−3465 to 8961)	285 856 (81 335–Dominated^b^)
Psychoeducation, depression effect	409.5 (386.6–432.2)	10 290 (7924–12 669)	Not applicable	Not applicable	Dominated^b^
Cognitive–behavioural therapy, depression effect	978.7 (931.0–1027.9)	8241 (4289–11 842)	Not applicable	Not applicable	Dominated^b^

UI, uncertainty intervals.

a A strategy is dominant when it provides more QALYs gained at a lower cost.

b A strategy is dominated when it provides fewer QALYs gained at a higher cost.

**Fig. 1:**
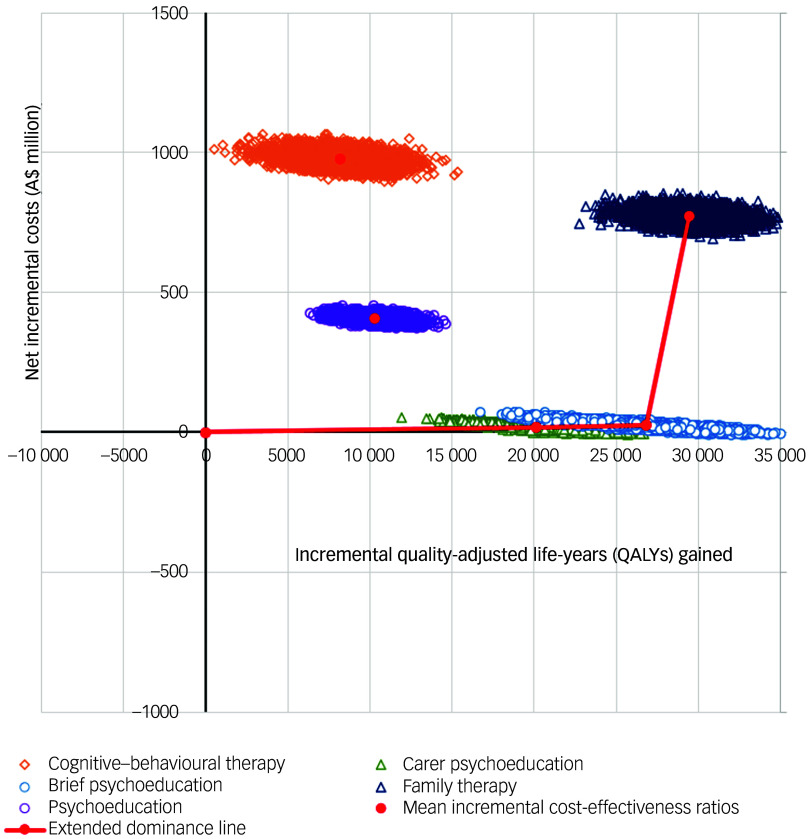
Cost-effectiveness plane for base-case analyses using quality-adjusted life-years (QALYs) gained as the outcome measure. The cost-effectiveness plane displays simulations across a four-quadrant graph by the difference in cost and the difference in outcomes. Points falling in the upper right quadrant represent a scenario where the intervention is more costly and more effective. Points falling in the lower right quadrant represent the scenario where the intervention is less costly and more effective, also known as dominant. While not shown here, any iterations falling in the lower left quadrant would be indicative of an intervention being less costly and less effective than the comparator. Any points falling in the upper left quadrant would be indicative of the intervention being more costly and less effective than the comparator, also referred to as the intervention being dominated.

Figure 1 provides a cost-effectiveness plane with simulations plotted for all interventions from the base-case probabilistic sensitivity analyses. The red line designates the cost-effectiveness frontier comprised of simulations from carer psychoeducation, brief psychoeducation and family therapy. Psychoeducation and CBT are dominated, with all simulations positioned to the left of the cost-effectiveness frontier indicating higher cost and fewer QALY gains.

**Fig. 2:**
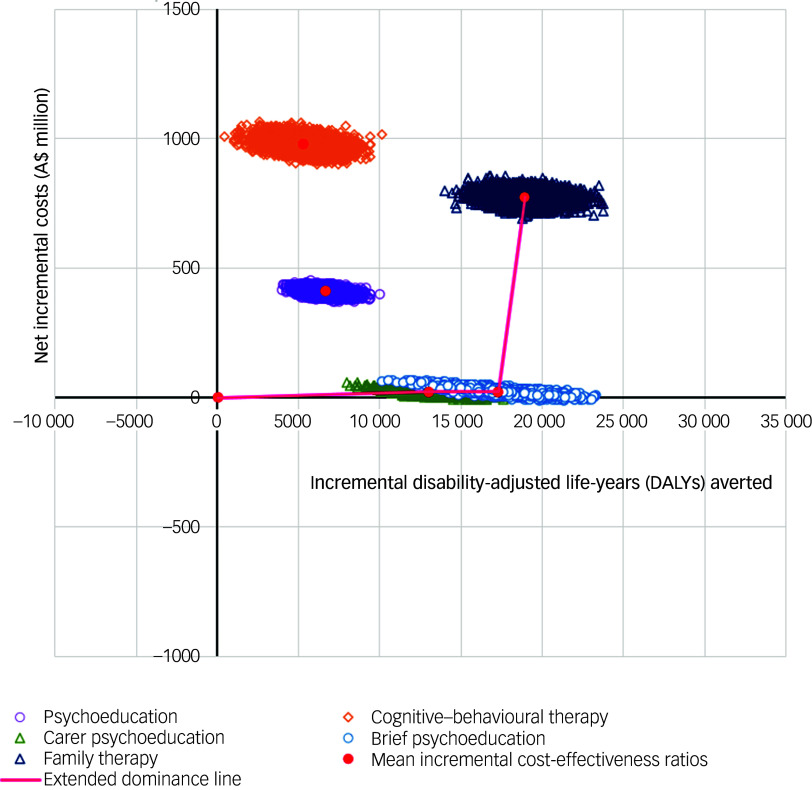
Cost-effectiveness plane for base-case analyses using disability-adjusted life-years (DALYs) averted as the outcome measure. The cost-effectiveness plane displays simulations across a four-quadrant graph by the difference in cost and the difference in outcomes. Points falling in the upper right quadrant represent a scenario where the intervention is more costly and more effective. Points falling in the lower right quadrant represent the scenario where the intervention is less costly and more effective, also known as dominant. While not shown here, any iterations falling in the lower left quadrant would be indicative of an intervention being less costly and less effective than the comparator. Any points falling in the upper left quadrant would be indicative of the intervention being more costly and less effective than the comparator, also referred to as the intervention being dominated.

**Fig. 3:**
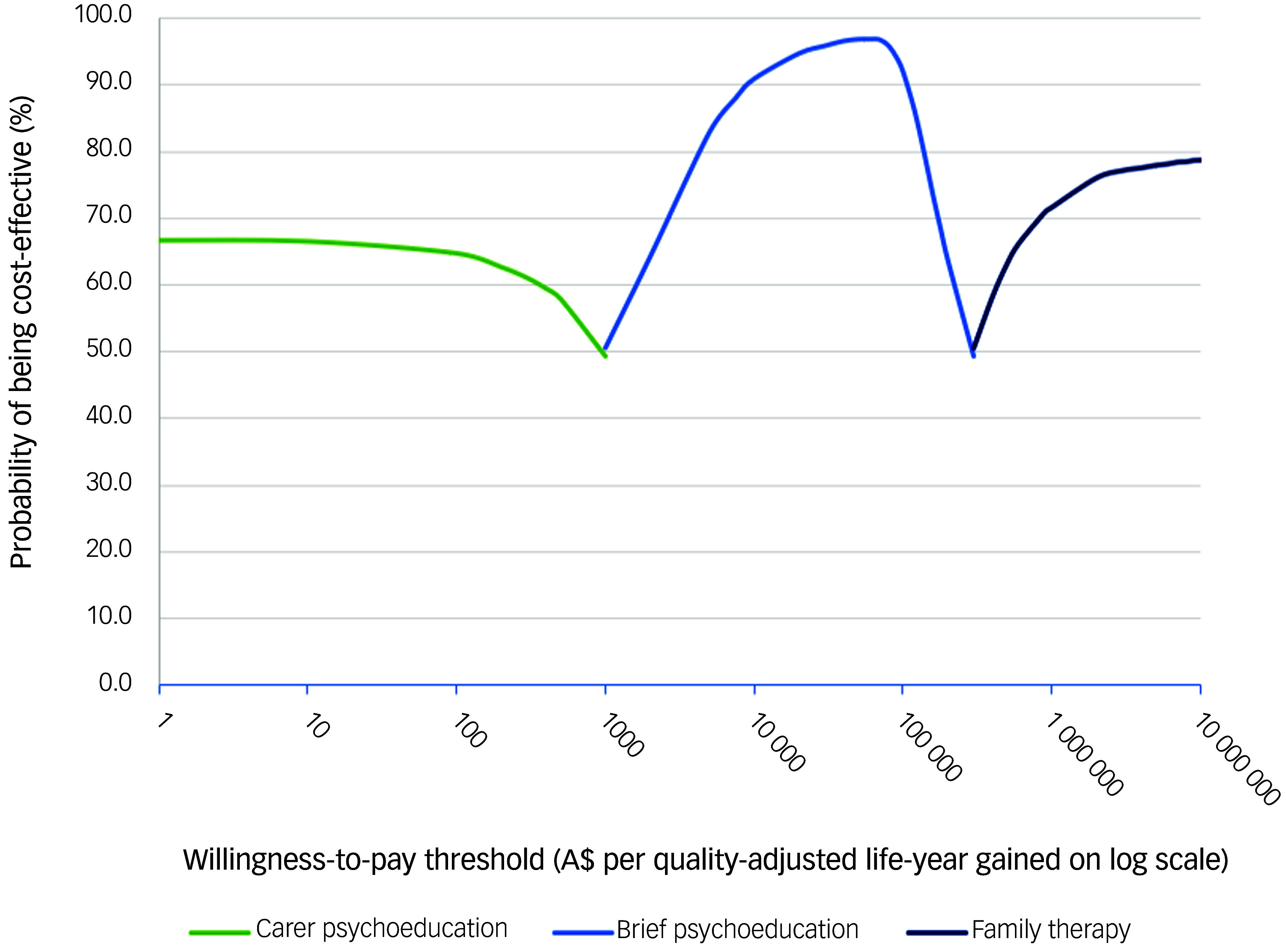
Cost-effectiveness acceptability frontier for base case analyses using quality-adjusted life-years (QALYs) gained as the outcome measure. This acceptability frontier presents the percentage of simulations falling below a range of willingness to pay thresholds on a log scale.

**Fig. 4:**
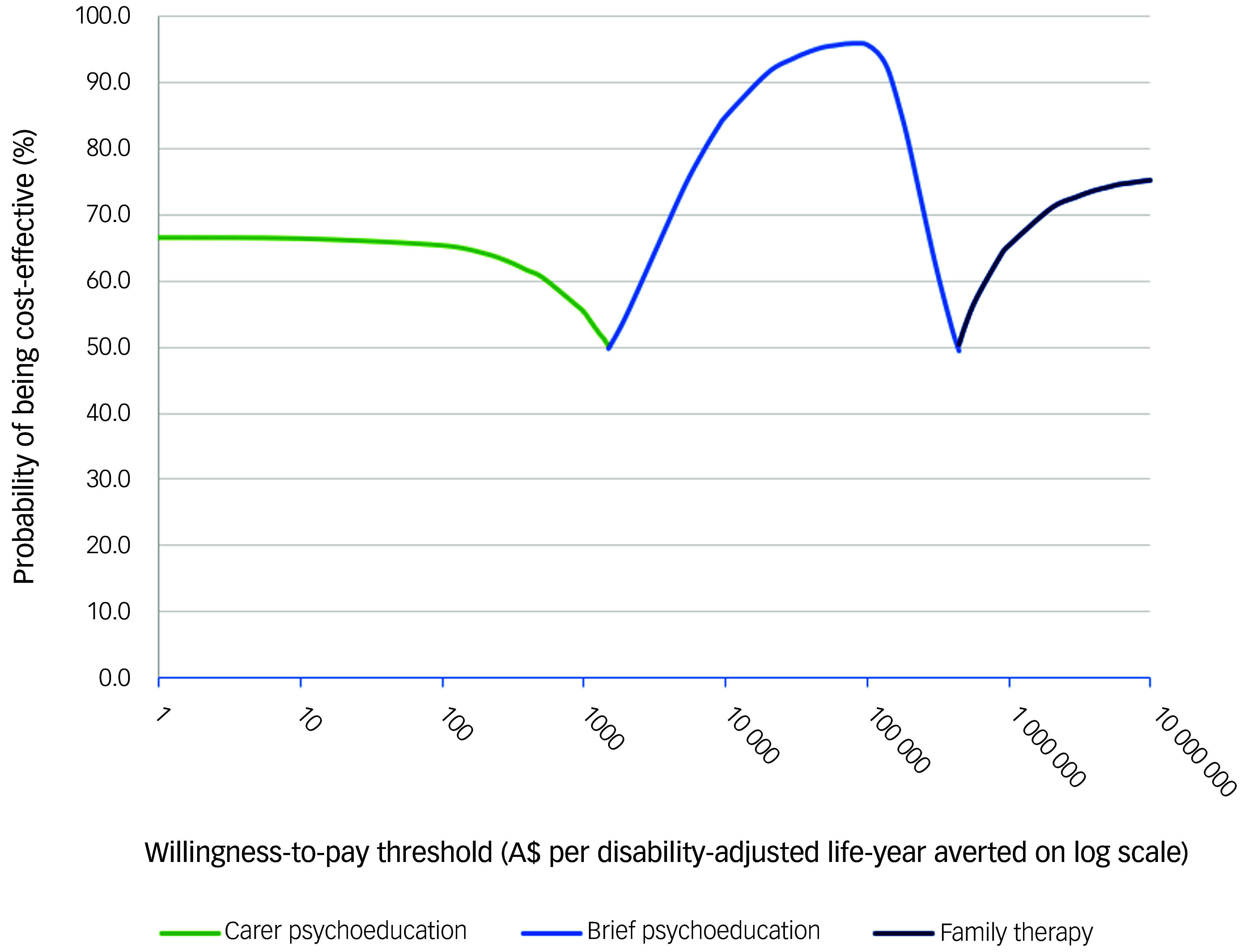
Cost-effectiveness acceptability frontier for base case analyses using diability-adjusted life-years (DALYs) averted as the outcome measure. This acceptability frontier presents the percentage of simulations falling below a range of willingness to pay thresholds on a log scale.

As depicted in the cost-effectiveness acceptability curves in Supplementary Appendix 1, Fig. 1, and the cost-effectiveness acceptability frontier in Fig. 2, carer psychoeducation is the preferred psychosocial therapy when the WTP threshold is below A$1000 per QALY gained. Brief psychoeducation would be preferred when the WTP is above A$1000 and below A$300 000 per QALY gained. Brief psychoeducation had>95% of simulations falling below the A$33 000 per QALY gained WTP threshold. Family therapy would be the preferred therapy only when the WTP threshold is above A$300 000 per QALY gained.

The ICER results for DALYs averted shown in Table 4, Fig. 3 and Supplementary Appendix 1, Fig. 2, follow a similar pattern to the QALY results. The cost-effectiveness acceptability curves (Supplementary Appendix 1, Fig. 2) and the cost-effectiveness frontier in Fig. 4, show that carer psychoeducation is the preferred psychosocial therapy at WTP thresholds below A$1500 per DALY averted. Brief psychoeducation would be preferred between WTP thresholds of A$1500 and A$450 000 per DALY averted. Family therapy is preferred only when the WTP threshold is above A$450 000 per DALY averted.

**Table 4:** Results of base-case analyses for the outcome of disability-adjusted life-years (DALYs) averted

Intervention	Total costs (A$ 2021, millions)	Total DALYs	Incremental costs (A$ 2021)	Incremental DALYs	Incremental cost per DALY averted (A$ 2021)
	Mean (95% UI)	Mean (95% UI)	Mean (95% UI)	Mean (95% UI)	Mean (95% UI)
Carer psychoeducation, depression effect	18.2 (1.9–36.0)	12 990(10 506–15 538)	–	–	–
Brief psychoeducation, depression effect	24.5 (2.6–48.5)	17 295(13 736–20 839)	6.35 (−21.0 to 35.9)	4304 (−70 to 8709)	1475 (298 613^a^–4121)
Family therapy, depression effect	776.3 (733.3–819.9)	18 979(16 361–21 712)	751.8 (700.5–801.3)	1684 (−2763 to 6229)	446 437 (128 621–Dominated^b^)
Psychoeducation, depression effect	409.5 (386.6–432.2)	6645 (5089–8282)	Not applicable	Not applicable	Dominated^b^
Cognitive–behavioural therapy, depression effect	978.7 (931.0–1027.9)	5323 (2748–7751)	Not applicable	Not applicable	Dominated^b^

UI, uncertainty interval.

a Result is the result of lower cost and fewer DALYs.

b A strategy is dominated when it provides fewer DALYs averted at a higher cost.

### Sensitivity analyses

The sensitivity analysis applying the relative risk of relapse to the mania phase (Tables 5 and 6; Supplementary Appendix 1, Figs 3–8) resulted in brief psychoeducation achieving cost-savings, the lowest total cost (−A$130.2 million, 95% UI −A$168.7 to −A$87.8) and estimated QALYs gained of 10 958 (95% UI 8765–13 235) and DALYs averted of 17 527 (95% UI 14 411–20 530). Carer psychoeducation achieved cost-savings, but was dominated by brief psychoeducation with higher total costs (−A$108.6 million, 95% UI −A$143.8 to −A$71.4) and fewer QALYs gained (8711, 95% UI 6823–10 669) and DALYs averted (13 968, 95% UI 11 323–16 682). Family therapy had higher total costs (A$592.5 million, 95% UI A$545.7–641.7) and delivered more QALYs gained, with an ICER of A$455 264 (95% UI A$169 947 to Dominated) and A$284 771 (95% UI A$119 558 to Dominated) per DALY averted compared with brief psychoeducation. Psychoeducation and CBT were both dominated since they had higher costs and fewer QALYs gained and DALYs averted compared with the three alternative therapies. The cost-effectiveness acceptability frontier curves in Supplementary Appendix 1, Figs 7 and 8, show that brief psychoeducation is the preferred psychosocial therapy in this sensitivity analysis when the WTP threshold is below A$450 000 per QALY gained and A$280 000 per DALY averted. Family therapy would be the preferred therapy when the WTP threshold is above those values.

**Table 5:** Results of sensitivity analyses for the outcome of quality-adjusted life-years (QALYs) gained

Intervention	Total costs (A$ 2021, millions)	Total QALYs	Incremental costs (A$ 2021)	Incremental QALYs	Incremental cost per QALY gained (A$ 2021)
	Mean (95% UI)	Mean (95% UI)	Mean (95% UI)	Mean (95% UI)	Mean (95% UI)
Brief psychoeducation, mania effect	−130.2 (−168.7 to −87.8)	10 958(8765–13 235)	–	–	–
Family therapy, mania effect	592.5 (545.7–641.7)	12 545(10 552–14 656)	722.7 (658.2–784.3)	1587 (–1528 to 4615)	455 388 (169 947–Dominated^a^)
Carer psychoeducation, mania effect	−108.6 ( −143.8 to −71.4)	8711(6823–10 669)	Not applicable	Not applicable	Dominated^a^
Psychoeducation, mania effect	343.0 (311.2–374.9)	4,477 (3334–5666)	Not applicable	Not applicable	Dominated^a^
Cognitive–behavioural therapy (CBT), mania effect	925.6 (862.6–987.9)	3,580 (1770–5379)	Not applicable	Not applicable	Dominated^a^
Brief psychoeducation, depression effect, delivered by psychologist	−18.0 (−35.2 to 1.5)	25 296(21 046–29 345)	–	–	–
Family therapy, depression effect, delivered by psychologist	493.6 (462.1–524.4)	28 901(25 586–32 158)	511.6 (474.7–548.0)	3604 (–1722 to 8953)	141 953 (61 212–Dominated^a^)
Carer psychoeducation, depression effect, delivered by psychologist	−6.4 (−22.3 to 11.1)	20 116(16 418–23 790)	Not applicable	Not applicable	Dominated^a^
Psychoeducation, depression effect, delivered by pschologist	278.4 (260.7–296.3)	10 331(8049–12 766)	Not applicable	Not applicable	Dominated^a^
CBT, depression effect, delivered by psychologist	665.4 (629.4–702.3)	8223 (4322–11 4934)	Not applicable	Not applicable	Dominated^a^

UI, uncertainty interval.

a A strategy is dominated when it provides fewer QALYs gained at a higher cost.

**Table 6:** Results of sensitivity analyses for the outcome of disability-adjusted life-years (DALYs) averted

Intervention	Total costs (A$ 2021, millions)	Total DALYs	Incremental costs (A$ 2021)	Incremental DALYs	Incremental cost per DALY averted (A$ 2021)
	Mean (95% UI)	Mean (95% UI)	Mean (95% UI)	Mean (95% UI)	Mean (95% UI)
Brief psychoeducation, mania effect	−130.2 (–168.7 to −87.8)	17 527(14 411–20 530)	–	–	–
Family therapy, mania effect	592.5 (545.7–641.7)	20 065(17 422–22 662)	722.7 (658.2–784.3)	2538 (−1489 to 6560)	284 771 (119 558–Dominated^a^)
Carer psychoeducation, mania effect	−108.6 (−143.8 to −71.4)	13 968(11 323–16 682)	Not applicable	Not applicable	Dominated^a^
Psychoeducation, mania effect	343.0 (311.2–374.9)	7153 (5472–8850)	Not applicable	Not applicable	Dominated^a^
Cognitive–behavioural therapy (CBT), mania effect	925.6 (862.6–987.9)	5736 (2989–8463)	Not applicable	Not applicable	Dominated^a^
Brief psychoeducation, depression effect, delivered by psychologist	−18.0 (–35.2 to 1.5)	16 330(13 354–19 243)	–	–	–
Family therapy, depression effect, delivered by psychologist	493.6 (462.1–524.4)	18 648(16 094–21 338)	511.6 (474.7–548.0)	2318 (–1,563 to 6378)	220 708 (85 933–Dominated^a^)
Carer psychoeducation, depression effect, delivered by psychologist	−6.4 (–22.3 to 11.1)	12 994(10 480–15 538)	Not applicable	Not applicable	Dominated^a^
Psychoeducation, depression effect, delivered by psychologist	278.4 (260.7–296.3)	6665 (5104–8307)	Not applicable	Not applicable	Dominated^a^
CBT, depression effect, delivered by psychologist	665.4 (629.4–702.3)	5316 (2819–7777)	Not applicable	Not applicable	Dominated^a^

UI, uncertainty interval.

a A strategy is dominated when it provides fewer DALYs averted at a higher cost.

The sensitivity analysis involving intervention delivery by a psychologist rather than a clinical psychologist (at a lower session cost) led to similar results with brief psychoeducation being the preferred psychosocial therapy up to a WTP threshold of A$140 000 per QALY gained and A$225 000 per DALY averted (Tables 5 and 6; Supplementary Appendix 1, Figs 9–14).

The sensitivity analysis extending the benefits of CBT to 5 years estimated 22 404 QALYs gained (Suppelementary Appendix 1, Table 8) and 14 325 DALYs averted (Supplementary Appendix 1, Table 9). CBT would remain dominated because these values are well below the outcomes for family therapy from the base case. In the scenario where psychoeducation was delivered through group sessions with a clinical psychologist, the total costs decreased by A$254.5 million, but with similar QALYs gained and DALYs averted, the intervention remained dominated (more costly and less effective) by brief psychoeducation.

## Discussion

This study found that carer psychoeducation was the most cost-effective adjunctive psychosocial therapy for treating bipolar disorder in Australian adults, when compared with four competing adjunctive therapies at WTP thresholds of A$1000 per QALY gained or A$1500 per DALY averted. Brief psychoeducation was found to be the preferred adjunctive therapy, in the base case, when the WTP threshold was between A$1000 and A$300 000 per QALY gained and between A$1500 and A$450 000 per DALY averted. Brief psychoeducation also had over 95% probability of cost-effectiveness relative to the A$33 000 per QALY gained or DALY averted WTP thresholds in the base-case analyses. In the sensitivity analyses, brief psychoeducation was the preferred intervention, with the potential for large cost savings. However, it is important to consider that this psychosocial therapy was a control condition in three trials of family therapy, and two trials focused on adolescents.^18^ Family therapy would only be considered the preferred adjunctive psychosocial therapy above a WTP threshold of A$300 000 per QALY gained or A$450 000 per DALY averted. Psychoeducation and CBT would not be considered cost-effective alternatives, given that they were dominated in all analyses.

The results of this economic evaluation suggest that psychoeducation should be available to all family members of people with bipolar disorder. MBS items are now available in Australia to provide subsidies for up to two services per calendar year to a carer/family member to be involved in an individual’s mental healthcare.^34^ However, these sessions count toward the maximum number of reimbursed sessions of the person with the mental health diagnosis, which is quite limited with a current cap of ten. This represents a significant health policy gap.

Our findings suggest that brief psychoeducation (three 1-h sessions) should be available to all people with bipolar disorder. In Australia, individuals with bipolar disorder would be able to receive psychoeducation as part of individual consultations or group sessions with psychiatrists, psychologists or allied health professionals partially subsidised through MBS under the Better Access initiative. The number of subsidised sessions is currently capped at ten per year, and the three sessions of brief psychoeducation would fit within this limit. There are, however, additional barriers to accessing brief psychoeducation, including the limited availability of mental health providers^35^ and out-of-pocket costs for sessions.^36^ There is also no specifically mandated regimen of psychoeducation, and currently no way to know the specific type of care being provided (i.e. psychoeducation, CBT, etc) as these details are not recorded in administrative data. Previous research showed that only 49% of individuals with affective disorders received an evidence-based psychological intervention.^37^

Our finding that 14 sessions of psychoeducation were not cost-effective may be viewed as controversial, given that current clinical guidelines recommend psychoeducation as first-line adjunctive treatment within the maintenance phase of bipolar disorder.^5^

These findings are comparable to a within-trial economic evaluation of structured group psychoeducation.^11^ That study found group psychoeducation was associated with additional costs and small QALY gains compared with unstructured group support, with a 35% probability of being cost-effective at a WTP threshold of £30 000 per QALY. Economic modelling from the same study estimated the probability of psychoeducation being cost-effective compared with TAU was 54% under the most extreme assumptions (probability of relapse and time to relapse was 20% higher for TAU).

Our results contrast with a previous modelled economic evaluation that found community-delivered group-based psychoeducation plus lithium or valproate was the most cost-effective strategy relative to hospital-based delivery or medication alone.^9^ The average ICER in the Western Pacific region, including Australia, was estimated at 20 000–30 000 International Dollars (approximately A$30 000–44 000) per DALY averted despite much higher disability weights used for the bipolar health states (manic 0.72, depressed 0.76 and interim 0.14) and more time spent in the depression health state for the ‘no intervention’ comparator.

The results for family therapy being cost-effective at WTP thresholds above A$300 000 per QALY gained or A$450 000 per DALY averted supports clinical guidelines that recommend family-focused therapy as second-line treatment for both the maintenance and depressive phases.^5^ Family group therapy is currently subsidised in Australia under the MBS, through general practitioner and psychiatrist item numbers. However, the potential out-of-pocket costs and ten session cap on Better Access funded mental health sessions^36^ may make this therapy inaccessible to many families. It is important to note that two of the seven studies included in the NMA for the effect size of family therapy were conducted on adolescents. Given the greater likelihood of family support available to adolescents and young adults, our results may be optimistic when applied across all adults with bipolar disorder.

Our findings that CBT would not be a cost-effective alternative to other adjunctive psychosocial therapies is a controversial finding given that clinical guidelines recommend CBT as a second-line therapy for bipolar disorder. Our finding was strongly influenced by the negative results for recurrence observed in the largest CBT study on this topic.^38^ It also contrasts with a previous trial-based economic evaluation which found that participants receiving cognitive therapy added to medication had better outcomes when compared with standard care, with costs offsets attributable to reductions in other service use.^8^ The cost of cognitive therapy in Lam et al^8^ (2021 A$2741 converted using the EPPI-Centre Cost Converter) was comparable to the current analysis. However, the use of ‘days with bipolar episodes’ as the outcome measure contributes to the different findings within the current study, which used DALYs averted and QALYs gained.

Our finding for CBT was also a result of the non-significant relative risk of recurrence estimated from the NMA. We replicated a statistically significant odds ratio for CBT in the prevention of recurrence using a random-effects model, similar to Miklowitz et al.^18^ However, when the results were expressed as a relative risk, the result for CBT became non-significant. This is likely because of the odds ratio overestimating the relative risk when the outcome (i.e. recurrence of depression or mania) is a relatively common event.^39^

Our results also diverged from previous evaluations that have found CBT cost-effective for several other mental disorders within an Australian context (e.g. major depression, generalised anxiety disorder, panic disorder and bulimia nervosa).^33,40–42^ Our economic evaluation model used the risk of relapse to depression and mania as the measure of effectiveness; however, it does not capture the significant reductions in depressive symptoms associated with CBT.^18^ Therefore, these results are quite conservative with regards to estimated health gains. Given that in unipolar depression CBT is an effective therapy, this literature is weighted to the maintenance phase rather than the treatment of acute bipolar depression, which might disadvantage CBT in such analyses.

It should be noted that our economic evaluation estimated intervention costs and effect sizes based on data obtained from trials analysing in-person psychosocial therapies. Newer delivery methods such as online formats have the potential to decrease the cost of delivering psychosocial therapies; however, to date, only therapist-guided internet interventions for depression, anxiety, smoking cessation and alcohol consumption have been shown to be cost-effective.^43^ There is limited and mixed evidence supporting the use of internet-delivered interventions for people with bipolar disorders.^44,45^ Additional research is required to understand the implications of delivering online psychosocial therapies on their effectiveness and cost-effectiveness specifically for use in people with bipolar disorder.

Our cost–utility results should not be viewed in isolation, but considered alongside other decision-making criteria such as illness severity, disease prevalence or rarity, equity and available alternatives to adjust thresholds or guide deliberative processes used by decision makers.^46^ In this analysis of the adult Australian population with bipolar disorder seeking treatment, the relatively low prevalence of bipolar disorder and the limited treatment alternatives available should be considered.

### Strengths and limitations

A strength of this analysis is the use of both DALYs averted and QALYs gained as outcome measures. Both QALYs and DALYs are composite measures of morbidity and mortality; however, their use in economic evaluation is varied. DALYs are an accepted measure of disease burden globally, but their use in economic evaluations has been limited to priority setting studies.^9,12^ This is largely because of the consistency in derivation of disability weights across multiple conditions allowing comparability of economic evaluations utilising DALYs. QALYs are more commonly required by health technology assessment agencies (i.e. NICE, PBAC) as the outcome measure in economic evaluations. However, the QALY outcomes utilised in this modelled analysis were limited because of the reliance on utility values from a relatively small cohort of individuals (<100) with bipolar disorder being treated in the USA almost 20 years ago.^29^

Our economic model did not account for the heterogeneity in disease course or adverse events from treatment. However, it is unlikely that the psychosocial therapies evaluated would be associated with adverse events requiring application of disutilities. The model did not account for potential changes to medication adherence, which could improve with interventions such as psychoeducation,^15^ leading to increased medication costs. However, there is limited evidence to quantify this effect. The model also did not account for treatment non-adherence, making a conservative assumption that all psychosocial sessions were provided and contributed to intervention effectiveness. The model excluded the cost of treating future ‘unrelated’ disease as well as production gains and losses and other non-health sector impacts. The model largely focuses on relapse outcomes for depressive or manic states, an important treatment aim in bipolar disorder. However, it did not capture reductions in manic or depressive symptoms and improved functioning from psychosocial therapy that are also meaningful outcomes to people living with bipolar disorder. There is some evidence to suggest that CBT is associated with stabilising depression symptoms,^18^ and the combination of psychoeducation and CBT is associated with improving symptoms of mania.^17^

In summary, carer and brief psychoeducation are potentially cost-effective adjunctive therapies for adults with bipolar disorder, and should be easily accessible to all Australians. Psychoeducation, family therapy and CBT are not cost-effective adjunctive therapies for adults with bipolar disorder, based on our results. Future research into the cost-effectiveness of these interventions based on symptom improvement is needed.

## Supporting information

Chatterton et al. supplementary material Chatterton et al. supplementary material

## Data Availability

Data supporting the findings of this study can be found in Tables 1 and 2 of the manuscript, in addition to Supplementary Tables 2–7. The data are also available on request from the corresponding author (M.L.C.).

## Supplementary material

The supplementary material is available online at https://doi.org/10.1192/bjo.2025.10068
