# Supplementary material for: Cost–utility analysis of adjunctive psychosocial therapies in bipolar disorder
Source: BJPsych Open. 2025 Jul 21;11(4):e156. doi: 10.1192/bjo.2025.10068 (PMC12303828; doi:10.1192/bjo.2025.10068)
Supplement: Chatterton et al. supplementary material [file S2056472425100689sup001.pdf]

Cost-utility analysis of adjunctive psychosocial therapies in bipolar disorder

Supplementary appendix

Table S1. Odds ratio results of re-analysis of Miklowitz et al. network meta-analysis in MetaXL using random effect model

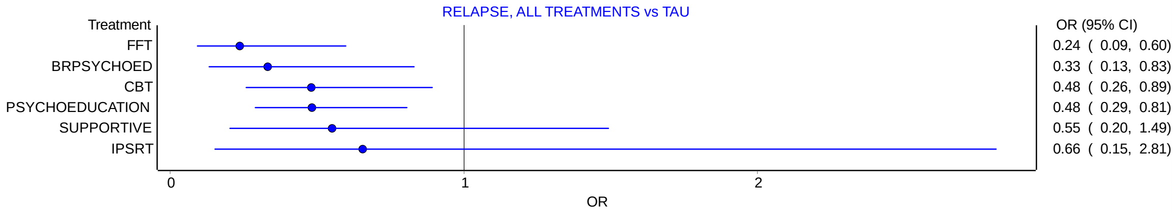

ID	Comparison	Active	Control	OR	LCI 95%	HCI 95%
	Direct estimates					
1	CBT-TAU	CBT	TAU	0.52	0.27	1.01
2	CBT-SUPPORTIVE	CBT	SUPPORTIVE	1.41	0.55	3.63
3	CBT-PSYCHOED	CBT	PSYCHOEDUCATION	0.63	0.12	3.43
4	PSYCHOED-TAU	PSYCHOEDUCATION	TAU	0.42	0.26	0.67
5	PSYCHOED-SUPPORTIVE	PSYCHOEDUCATION	SUPPORTIVE	0.40	0.10	1.60
6	FFT-TAU	FFT	TAU	0.29	0.15	0.56
7	FFT-PSYCHOED	FFT	PSYCHOEDUCATION	0.27	0.08	0.84
8	FFT-BRPSYCHOED	FFT	BRPSYCHOED	0.88	0.46	1.67
9	IPSRT-SUPPORTIVE	IPSRT	SUPPORTIVE	1.77	0.73	4.31
	Indirect estimates (source IDs)					
10	Indirect CBT vs TAU (3, 4)	CBT	TAU	0.26	0.05	1.53
11	Indirect FFT vs TAU (7, 4)	FFT	TAU	0.11	0.03	0.39
12	Indirect SUPPORTIVE vs TAU (2, 1)	SUPPORTIVE	TAU	0.37	0.12	1.17
13	Indirect PSYCHOEDUCATION vs TAU (3, 1)	PSYCHOEDUCATION	TAU	0.83	0.13	5.09
14	Indirect SUPPORTIVE vs TAU (5, 4)	SUPPORTIVE	TAU	1.04	0.24	4.50
15	Indirect PSYCHOEDUCATION vs TAU (7, 6)	PSYCHOEDUCATION	TAU	1.10	0.29	4.11
16	Indirect BRPSYCHOED vs TAU (8, 6)	BRPSYCHOED	TAU	0.33	0.13	0.83
17	Extended Indirect IPSRT vs TAU (9, 12)	IPSRT	TAU	0.66	0.15	2.81
	Result estimates (source IDs)					

	CBT (1, 10)	CBT	TAU	0.48	0.26	0.89
	SUPPORTIVE (12, 14)	SUPPORTIVE	TAU	0.55	0.20	1.49
	PSYCHOEDUCATION (4, 13, 15)	PSYCHOEDUCATION	TAU	0.48	0.29	0.81
	FFT (6, 11)	FFT	TAU	0.24	0.09	0.60
	BRPSYCHOED (16)	BRPSYCHOED	TAU	0.33	0.13	0.83
	IPSRT (17)	IPSRT	TAU	0.66	0.15	2.81

Table S2. Relative risk of relapse/recurrence results of re-analysis of Miklowitz et al. network meta-analysis in MetaXL using random effect model

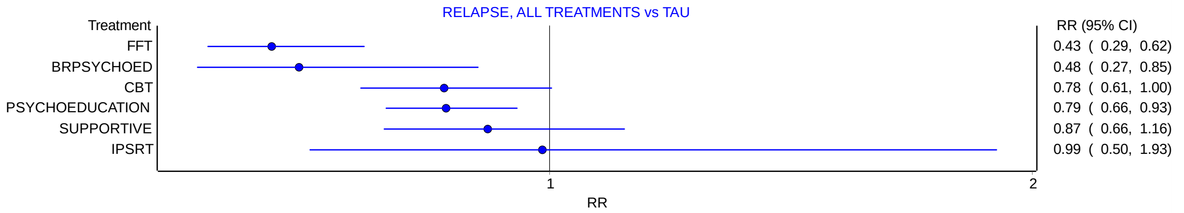

ID	Comparison	Active	Control	RR	LCI 95%	HCI 95%
	Direct estimates					
1	CBT-TAU	CBT	TAU	0.80	0.62	1.03
2	CBT-SUPPORTIVE	CBT	SUPPORTIVE	1.13	0.81	1.58
3	CBT-PSYCHOED	CBT	PSYCHOEDUCATION	0.67	0.21	2.15
4	PSYCHOED-TAU	PSYCHOEDUCATION	TAU	0.77	0.65	0.92
5	PSYCHOED-SUPPORTIVE	PSYCHOEDUCATION	SUPPORTIVE	0.81	0.66	0.99
6	FFT-TAU	FFT	TAU	0.45	0.29	0.70
7	FFT-PSYCHOED	FFT	PSYCHOEDUCATION	0.48	0.24	0.93
8	FFT-BRPSYCHOED	FFT	BRPSYCHOED	0.94	0.65	1.35
9	IPSRT-SUPPORTIVE	IPSRT	SUPPORTIVE	1.40	0.83	2.35
	Indirect estimates (source IDs)					
10	Indirect CBT vs TAU (3, 4)	CBT	TAU	0.52	0.16	1.68
11	Indirect FFT vs TAU (7, 4)	FFT	TAU	0.37	0.18	0.73
12	Indirect SUPPORTIVE vs TAU (2, 1)	SUPPORTIVE	TAU	0.71	0.46	1.08
13	Indirect PSYCHOEDUCATION vs TAU (3, 1)	PSYCHOEDUCATION	TAU	1.19	0.36	3.95
14	Indirect SUPPORTIVE vs TAU (5, 4)	SUPPORTIVE	TAU	0.95	0.73	1.25
15	Indirect PSYCHOEDUCATION vs TAU (7, 6)	PSYCHOEDUCATION	TAU	0.95	0.43	2.11
16	Indirect BRPSYCHOED vs TAU (8, 6)	BRPSYCHOED	TAU	0.48	0.27	0.85
17	Extended Indirect IPSRT vs TAU (9, 12)	IPSRT	TAU	0.99	0.50	1.93
	Result estimates (source IDs)					
	CBT (1, 10)	CBT	TAU	0.78	0.61	1.00
	SUPPORTIVE (12, 14)	SUPPORTIVE	TAU	0.87	0.66	1.16
	PSYCHOEDUCATION (4, 13, 15)	PSYCHOEDUCATION	TAU	0.79	0.66	0.93
	FFT (6, 11)	FFT	TAU	0.43	0.29	0.62
	BRPSYCHOED (16)	BRPSYCHOED	TAU	0.48	0.27	0.85
	IPSRT (17)	IPSRT	TAU	0.99	0.50	1.93

Table S3. Relative risk of relapse/recurrence results of re-analysis of Miklowitz et al. network meta-analysis in MetaXL using inverse variance heterogeneity model

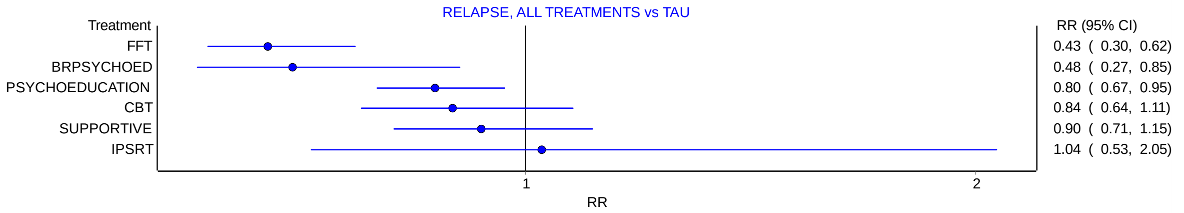

ID	Comparison	Active	Control	RR	LCI 95%	HCI 95%
	Direct estimates					
1	CBT-TAU	CBT	TAU	0.84	0.63	1.11
2	CBT-SUPPORTIVE	CBT	SUPPORTIVE	1.13	0.81	1.58
3	CBT-PSYCHOED	CBT	PSYCHOEDUCATION	1.05	0.24	4.51
4	PSYCHOED-TAU	PSYCHOEDUCATION	TAU	0.79	0.66	0.95
5	PSYCHOED-SUPPORTIVE	PSYCHOEDUCATION	SUPPORTIVE	0.81	0.66	1.00
6	FFT-TAU	FFT	TAU	0.45	0.29	0.70
7	FFT-PSYCHOED	FFT	PSYCHOEDUCATION	0.48	0.24	0.93
8	FFT-BRPSYCHOED	FFT	BRPSYCHOED	0.93	0.65	1.35
9	IPSRT-SUPPORTIVE	IPSRT	SUPPORTIVE	1.40	0.83	2.35
	Indirect estimates (source IDs)					
10	Indirect CBT vs TAU (3, 4)	CBT	TAU	0.83	0.19	3.62
11	Indirect FFT vs TAU (7, 4)	FFT	TAU	0.38	0.19	0.75
12	Indirect SUPPORTIVE vs TAU (2, 1)	SUPPORTIVE	TAU	0.74	0.48	1.15
13	Indirect PSYCHOEDUCATION vs TAU (3, 1)	PSYCHOEDUCATION	TAU	0.80	0.18	3.55
14	Indirect SUPPORTIVE vs TAU (5, 4)	SUPPORTIVE	TAU	0.97	0.74	1.28
15	Indirect PSYCHOEDUCATION vs TAU (7, 6)	PSYCHOEDUCATION	TAU	0.95	0.43	2.11
16	Indirect BRPSYCHOED vs TAU (8, 6)	BRPSYCHOED	TAU	0.48	0.27	0.85
17	Extended Indirect IPSRT vs TAU (9, 12)	IPSRT	TAU	1.04	0.53	2.05
	Result estimates (source IDs)					
	CBT (1, 10)	CBT	TAU	0.84	0.64	1.11
	SUPPORTIVE (12, 14)	SUPPORTIVE	TAU	0.90	0.71	1.15
	PSYCHOEDUCATION (4, 13, 15)	PSYCHOEDUCATION	TAU	0.80	0.67	0.95
	FFT (6, 11)	FFT	TAU	0.43	0.30	0.62
	BRPSYCHOED (16)	BRPSYCHOED	TAU	0.48	0.27	0.85
	IPSRT (17)	IPSRT	TAU	1.04	0.53	2.05

Table S4. Characteristics of psychosocial interventions included in network meta-analyses

Study	Facilitator	Session type	n	Number of sessions	Session length (mins)	Follow up (months)	Weighted average number of sessions[a]
**Psychoeducation**							13.5
Cardoso 2015[1]	Senior psychology students	Individual (assumed)	32	6	60	12	
Colom 2009[2]	Experienced psychologists (> 3 years' experience with bipolar patients)	Group	60	21	90	24	
Harvey 2015 (control group)[3]	Doctoral- or master's-level therapists.	Individual (assumed)	28	8	60	6	
Lobban 2010[4]	Care coordinators	Individual (assumed)	56	6	60	12	
Morris 2016[5]	Health professional and service user facilitators trained and supervised by a psychiatrist or clinical psychologist.	Group	153	21	120	24	
Parikh 2012 (control group)[6]	Experienced psychiatric staff (4 nurses, 2 psychotherapists and one psychiatrist) after brief training.	Group	109	6	90	18	
Perry 1999	Research psychologist with little previous clinical experience	Individual	33	12		12	
Rea 2003 (control group)[7]	Family therapists with training in Family focused treatment	Individual	25	21	30	24	
Reinares 2008[8]	Experienced psychologist	Group	57	12	90	12	
**Brief psychoeducation**							2.6

Miklowitz 2003[9]	19 therapists (3 doctoral, 14 master's and 2 with bachelor's degrees). All trained by lead author.	Individual family sessions	70	2	60	24	
Miklowitz 2008[10]	Clinicians receiving standardized training on the intervention during a 2-day pre-trial seminar.	Individual family sessions	28	3	50	24	
Miklowitz 2014[11]	Clinicians underwent training during a 2-day pretrial workshop and received monthly group teleconference supervision.	Individual family sessions	73	3	50	24	
**Carer psychoeducation**							8.3
Bordbar 2009[12]	Psychiatrist.	Group	29	1	120	12	
Reinares 2008[8]	Experienced psychologist.	Group	57	12	90	12	
**Cognitive behavioural therapy (CBT)**							20.2
Ball 2006[13]	Clinical psychologist.	Individual (assumed)	25	20	60	12	
Cochran 1984[14]	Author and graduate student with training.	Individual	14	6	60	6	
Gomes 2011[15]	Four clinical researchers formally trained in CBT with 5 years of experience.	Group	33	18	90	24	
Harvey 2015[3]	Doctoral- or master's-level therapists.	Individual (assumed)	30	8	60	6	
Jones 2015[16]	Mental health professionals trained to British Association for Behavioural and Cognitive Psychotherapies accreditation level in CBT.	Individual	33	18	60	12	

Lam 2000[17]	Clinical psychologists with a minimum of 6 years of postgraduate qualification experience.	Individual (assumed)	13	20	Not recorded	12	
Lam 2005[18]	Clinical psychologist with minimum of 5 years' experience.	Individual	51	20	60	6	
Meyer 2012[19]	Therapists had at least 1 year of postgraduate training in CBT and had attended a 2-day workshop about CBT and ST for bipolar disorder.	Individual	38	20	60	24	
Parikh 2012[6]	Master's degree (minimum) and one year of experience with CBT (2 psychologists, 2 psychiatrists, 7 psychotherapists, 2 nurses).	Individual	95	20	50	18	
Scott 2006[20]	Therapist with training in CBT and specific training in bipolar disorder.	Individual	127	26	Not recorded	24	
**Family therapy**							18.3
Bordbar 2009[12]	Psychiatrist	Group	29	1	120	12	
D'Souza 2010[21]	Four mental health clinicians, led and trained by the lead author	Group	20	12	90	12	
Nagy 2015[22]	Not stated	Individual family sessions (assumed)	67	21	60	12	

Miklowitz 2003[9]	19 therapists (3 doctoral, 14 master's and 2 with bachelor's degrees). All trained by lead author	Individual family sessions	31	21	60	24	
Miklowitz 2008[10]	Clinicians receiving standardized training on the intervention during a 2-day pre-trial seminar	Individual family sessions	30	21	50	24	
Miklowitz 2014[11]	Clinicians underwent training during a 2-day pretrial workshop and received monthly group teleconference supervision	Individual family sessions	72	21	50	24	
Rea 2003[7]	Family therapists with training in Family focused treatment	Individual	28	21	60	12	

[a] Calculated by multiplying the number of psychosocial sessions by the number of study participants in each study, summing these products and then dividing by the sum of the study participants across all studies.

Table S5. Intervention costs applied in the base case of the model

	Number	Unit cost[a]	% receiving	Total cost
**Psychoeducation**				
Group session with a clinical psychologist (MBS items 80020, 80021)	14	$38.95	50%	$545.30
Individual session with a clinical psychologist (MBS items 80010, 80011)	14	$135.97	50%	$1,903.58
Weighted average cost				$1,224.44
**Brief psychoeducation**				
Individual session with a clinical psychologist (MBS items 80010, 80011)	3	$135.97	100%	$407.91
**Carer psychoeducation**				
Group session with a clinical psychologist (MBS items 80020, 80021)	8	$38.95	100%	$311.60
**Cognitive behavioural therapy (CBT)**				
Individual session with a clinical psychologist (MBS items 80010, 80011)	20	$135.97	100%	$2,719.40
**Family therapy**				
Individual session with a clinical psychologist (MBS items 80010, 80011)	18	$135.97	100%	$2,447.46

[a]Derived from MBS item reports for July 2021 – June 2022[23]

Table S6. Resource use and cost of standard care by model health state in 2021 Australian Dollars

	Percentage requiring care type	Type of care	Number	Unit cost	Total cost
**Acute Mania/mixed episode**					
	60%	General practitioner	2	$39.10[a]	$78.20
	30%	Community Mental Health team	2	$264.41[b]	$528.83
	10%	Emergency department	1	$1,629.78[c,d]	$1,629.78
Subtotal detection of mania/mixed episode costs					$368.55[e]
	17.5%	Hospital admission (LOS = 13.47)	1	$23,076.48[c,d]	$23,076.48
	47.5%	Community Mental Health team (every other day)	12	$264.41[b]	$3,172.97
	25.0%	Ambulatory care by a general practitioner (every other day)	12	$39.10[a]	$469.20
	10.0%	Private psychiatrist (every other day)	12	$140.55[a]	$1,686.60
	5.0%	Private hospital stay (28 days)	1	$10,224.78[c,d]	$10,224.78
Subtotal treatment of mania/mixed episode costs					$6,342.75[e]
		**Total cost manic/mixed episode per person per year**			**$6,711.29**
**Acute depressive episode**					
	70.0%	General practitioner	2	$39.10[a]	$78.20
	30.0%	Community Mental Health team	4	$264.41[b]	$1,057.66
Subtotal detection of depressive episode costs[f]					$372.04[e]
	10.0%	10-day inpatient public hospital	10	$1,713.17[c,d]	$17,131.73
	10.0%	Community Mental Health team (once per week)	4	$264.41[b]	$1,057.66
	30.0%	General practitioner (once per week)	4	$39.10[a]	$156.40
	10.0%	Private psychiatrist (once per week)	4	$140.55[a]	$562.20
	2.5%	Private hospital stay (10 days)	10	$575.15[c,d]	$5,751.47
Subtotal treatment of depressive episode costs					$2,065.87[e]
		**Total cost depressive episode per person per year**			**$2,437.90**

[a]Medicare Benefits Schedule[24]; [b]expenditure for community mental health services[25] divided by the number of services[26]; [c]National Hospital Cost Data Collection[27];[d]inflated to 2021 prices using the health price index[28]; [e]Calculated by multiplying the percentage of the population requiring a service by the total cost of the service and then summing

these products. [f]Tolkien II[29] identified the use of clinical psychologists by 10% of the population in the acute depressive phase. This was removed from the costing to avoid double counting with the adjunctive psychosocial interventions being evaluated.

Table S7. Intervention costs applied in the sensitivity analysis using the cost of a psychologist

	Number	Unit cost[a]	% receiving	Total cost
**Psychoeducation**				
Group session with a psychologist (MBS items 80120, 80121)	14	$30.79	50%	$431.06
Individual session with a psychologist (MBS items 80110, 80111)	14	$94.24	50%	$1,319.36
Average cost				$875.21
**Brief psychoeducation**				
Individual session with a psychologist (MBS items 80110, 80111)	3	$94.24	100%	$282.72
**Carer psychoeducation**				
Group session with a psychologist (MBS items 80120, 80121)	8	$30.79	100%	$246.32
**Cognitive behavioural therapy (CBT)**				
Individual session with a psychologist (MBS items 80110, 80111)	20	$94.24	100%	$1,884.80
**Family therapy**				
Individual session with a psychologist (MBS items 80110, 80111)	18	$94.24	100%	$1,696.32

[a]Derived from MBS item reports for July 2021 – June 2022[23]

Table S8. Results of sensitivity analyses extending the effect of CBT for the outcome of QALYs gained

	Total costs (A$ 2021, millions) Mean (95% UI)	QALYs gained Mean (95% UI)	Average cost per QALY gained Mean (95% UI)
**CBT effect extended to 2 years**	$959.5 ($906.6 - $1,012.2)	12,333 (6,674 - 17,710)	$77,796 ($52,603 - $147,825)
**CBT effect extended to 3 years**	$949.5 ($894.4- $1,004.0)	14,457 (7,682 - 20,965)	$65,675 ($43,661 - $128,513)
**CBT effect extended to 4 years**	$928.9 ($869.2 - $990,7)	18,556 (10,271 - 26,711)	$50,060 ($33,423 - $94,301)
**CBT effect extended to 5 years**	$911.0 ($844.8 - $976.8)	22,404 (12,005 - 32,394)	$40,664 ($26,519 - $79,916)

Table S9. Results of sensitivity analyses extending the effect of CBT for the outcome of DALYs averted

	Total costs (A$ 2021, millions) Mean (95% UI)	DALYs averted Mean (95% UI)	Average cost per DALY averted Mean (95% UI)
**CBT effect extended to 2 years**	$959.5 ($906.6 - $1,012.2)	7,958 (4,169 - 11,564)	$120,571 ($80,846 - $237,469)
**CBT effect extended to 3 years**	$949.5 ($894.4- $1,004.0)	9,293 (4,944 - 13,545)	$102,164 ($67,672 - $200,599)
**CBT effect extended to 4 years**	$928.9 ($869.2 - $990,7)	11,927 (6,405 - 17,445)	$77,885 ($51,224 - $150,618)
**CBT effect extended to 5 years**	$911.0 ($844.8 - $976.8)	14,325 (7,702 - 21,021)	$63,596 ($41,324 - $124,829)

Figure S1. Cost-effectiveness acceptability curves for the base case using quality-adjusted life-years (QALYs) gained as the outcome measure

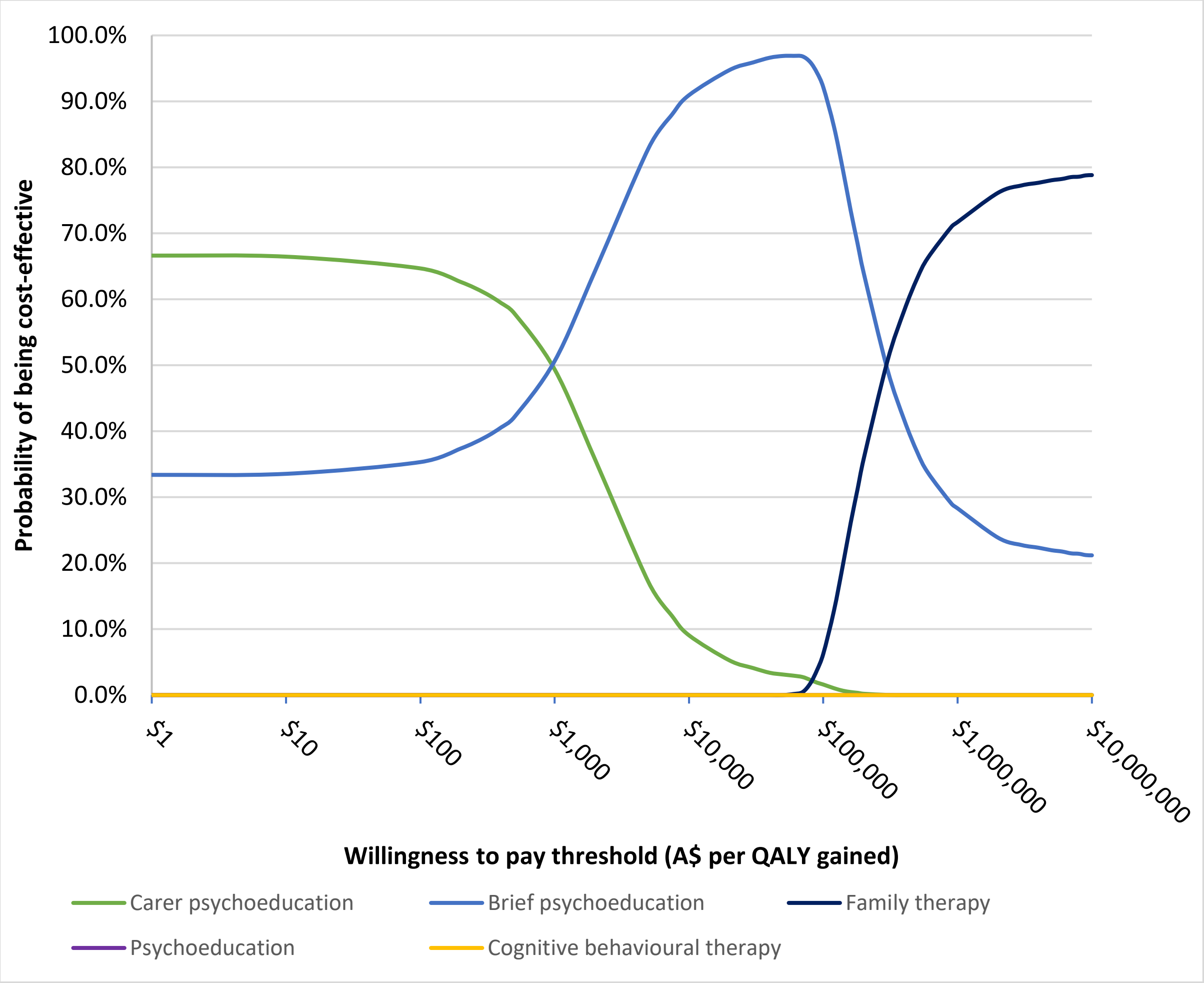

Figure S2. Cost-effectiveness acceptability curves for the base case analyses using disability-adjusted life-years (DALYs) averted as the outcome measure

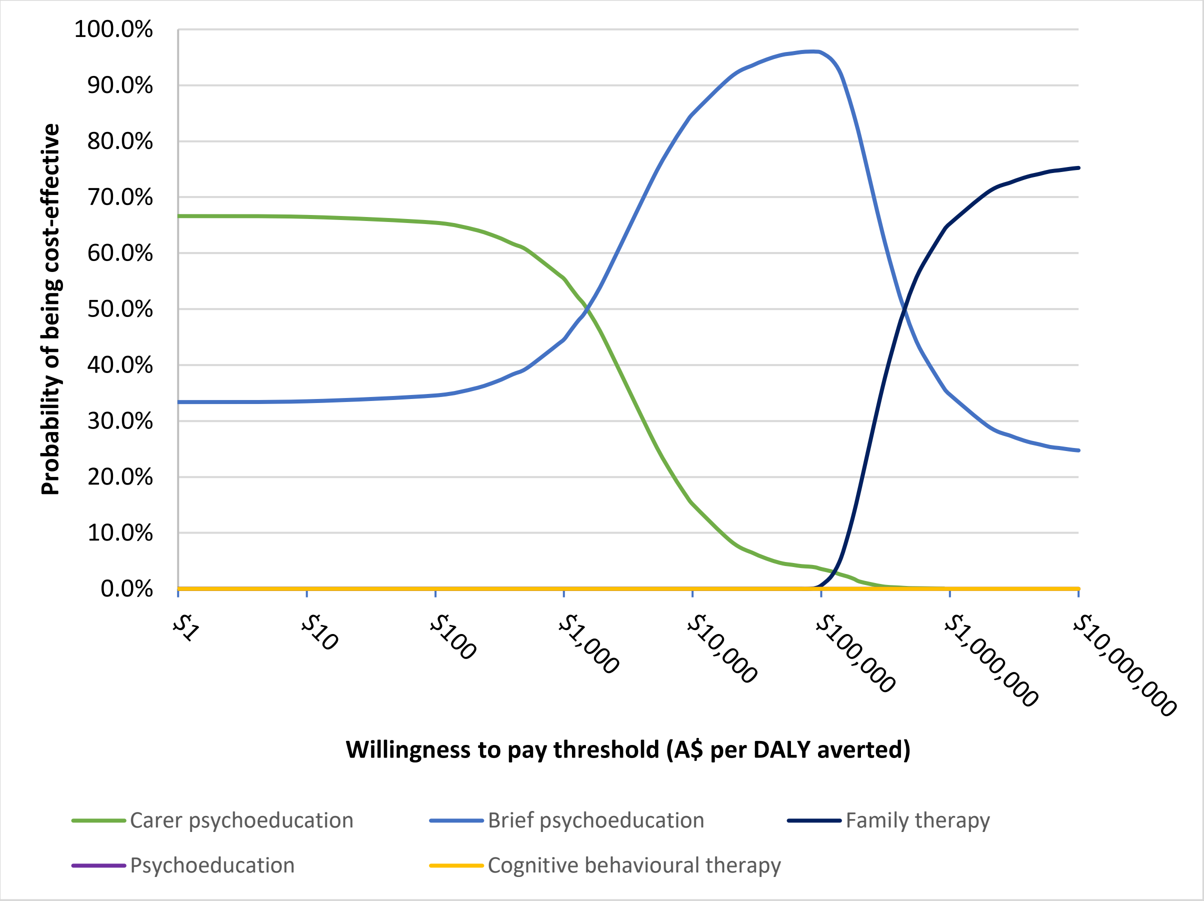

Figure S3. Cost-effectiveness plane for sensitivity analyses applying the effect to the mania phase using quality-adjusted life-years (QALYs) gained as the outcome measure

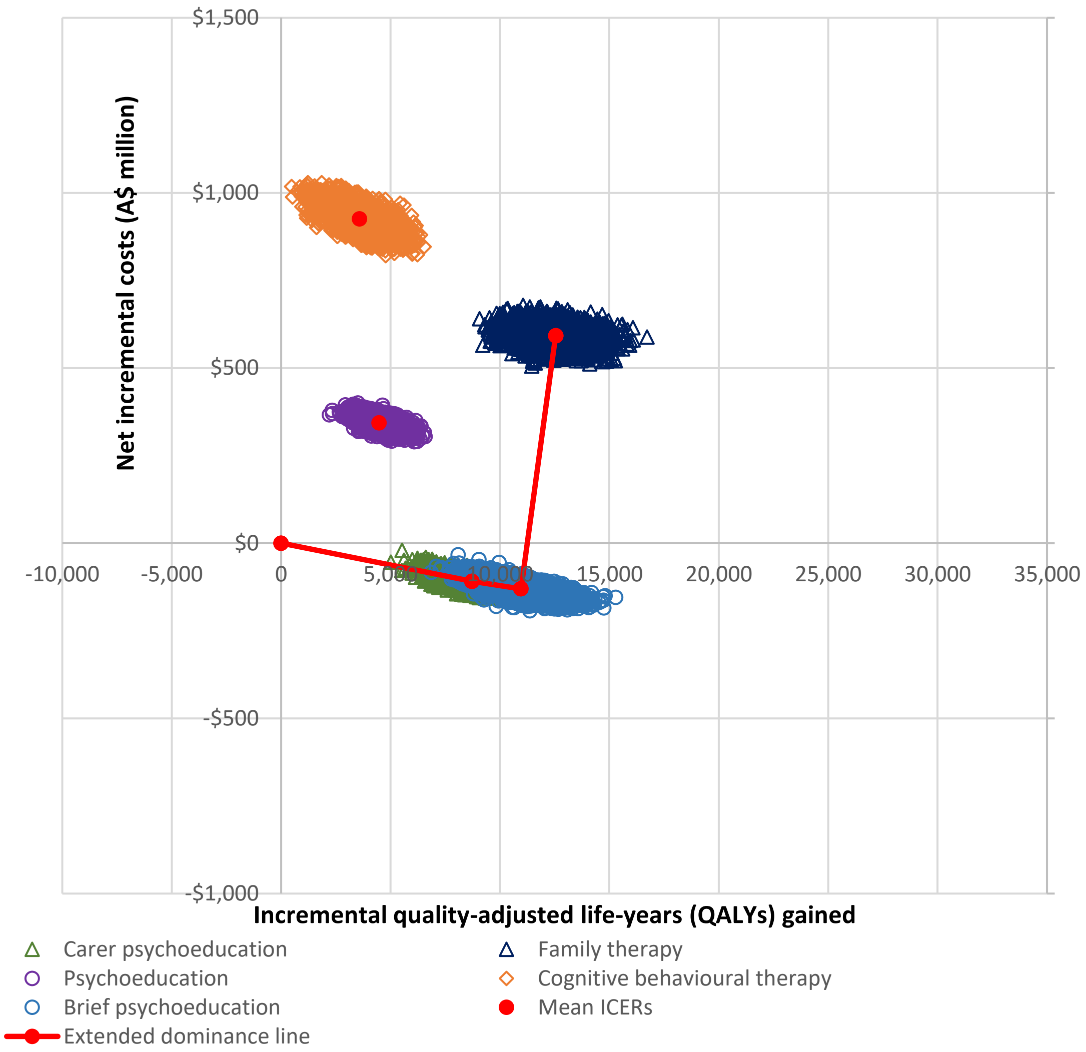

Figure S4. Cost-effectiveness plane for sensitivity analyses applying the effect to the mania phase using disability-adjusted life-years (DALYs) averted as the outcome measure

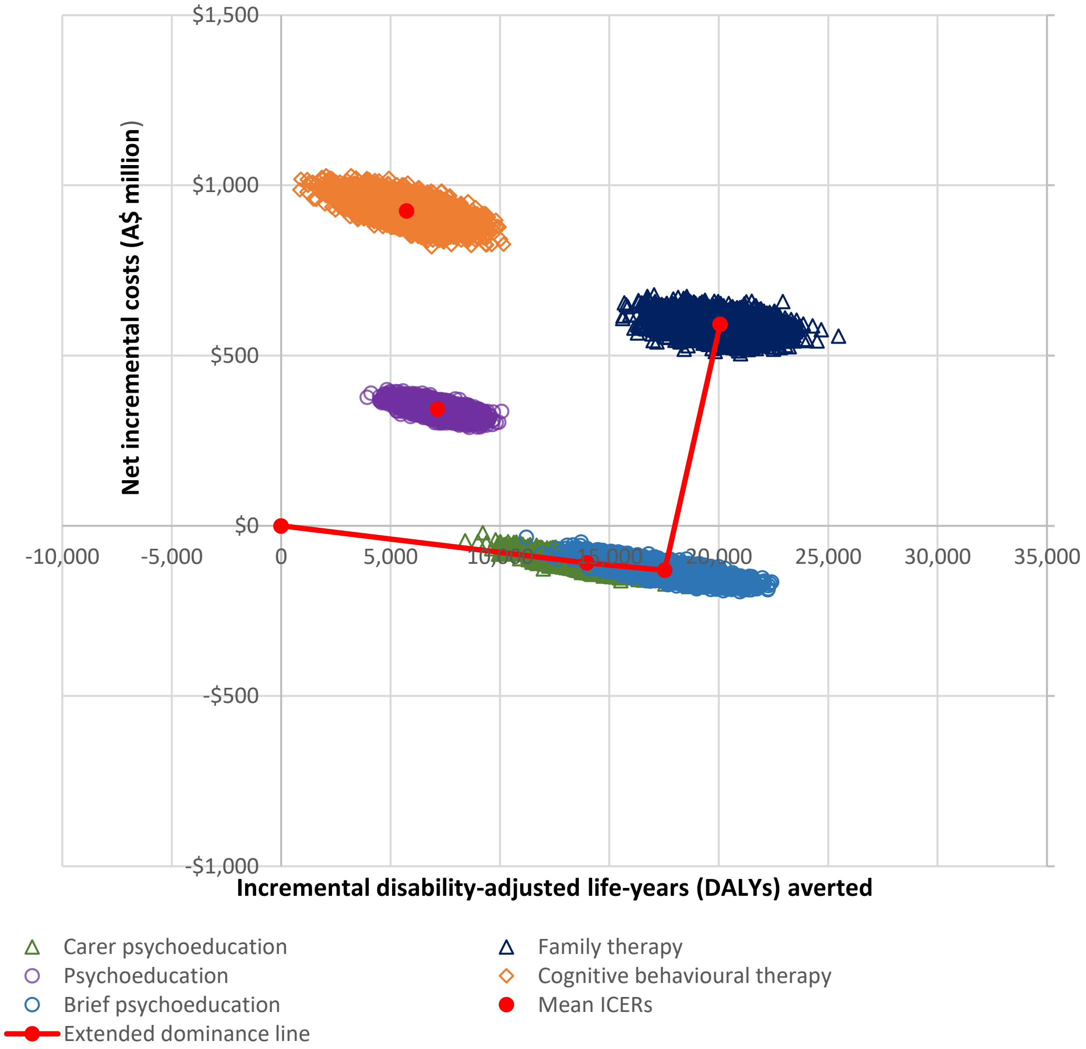

Figure S5. Cost-effectiveness acceptability curves for the sensitivity analyses applying the effect to the mania phase using quality-adjusted life-years (QALYs) gained as the outcome measure

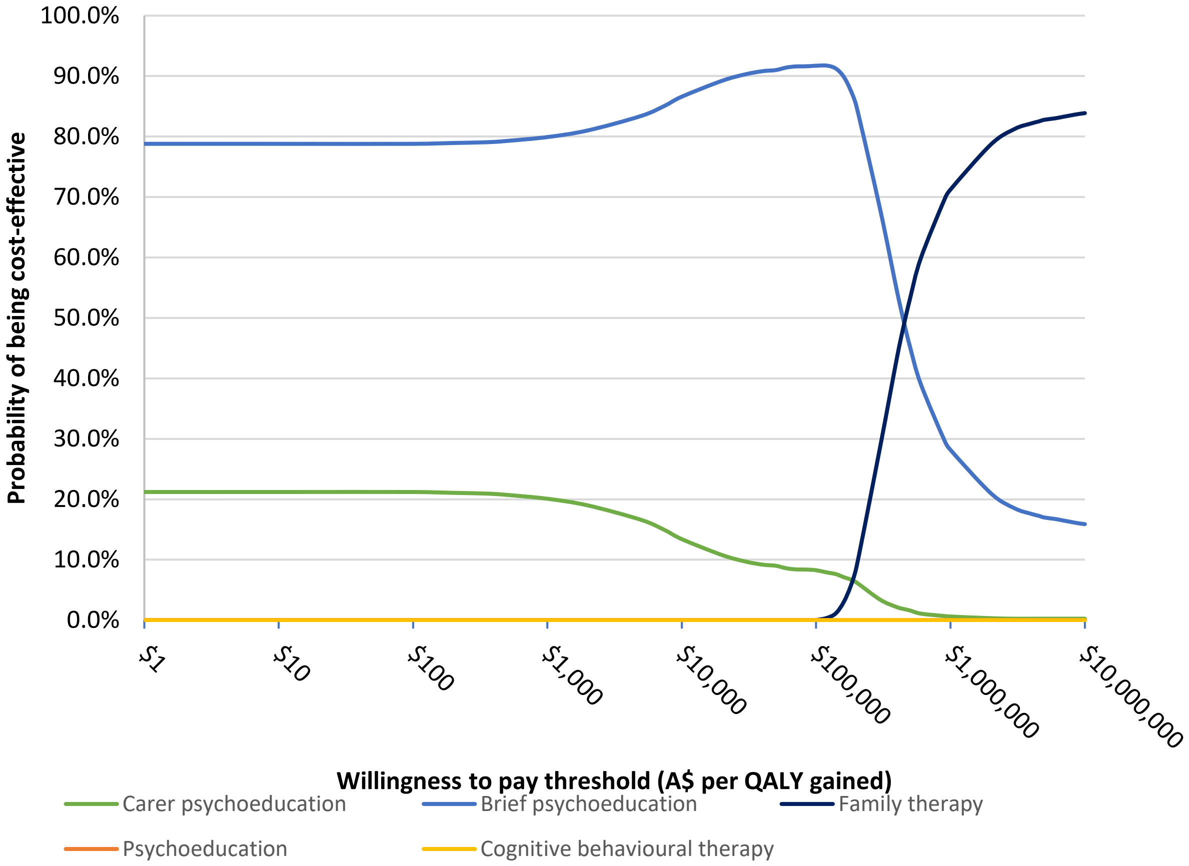

Figure S6. Cost-effectiveness acceptability curves for the sensitivity analyses applying the effect to the mania phase using disability-adjusted life-years (DALYs) gained as the outcome measure

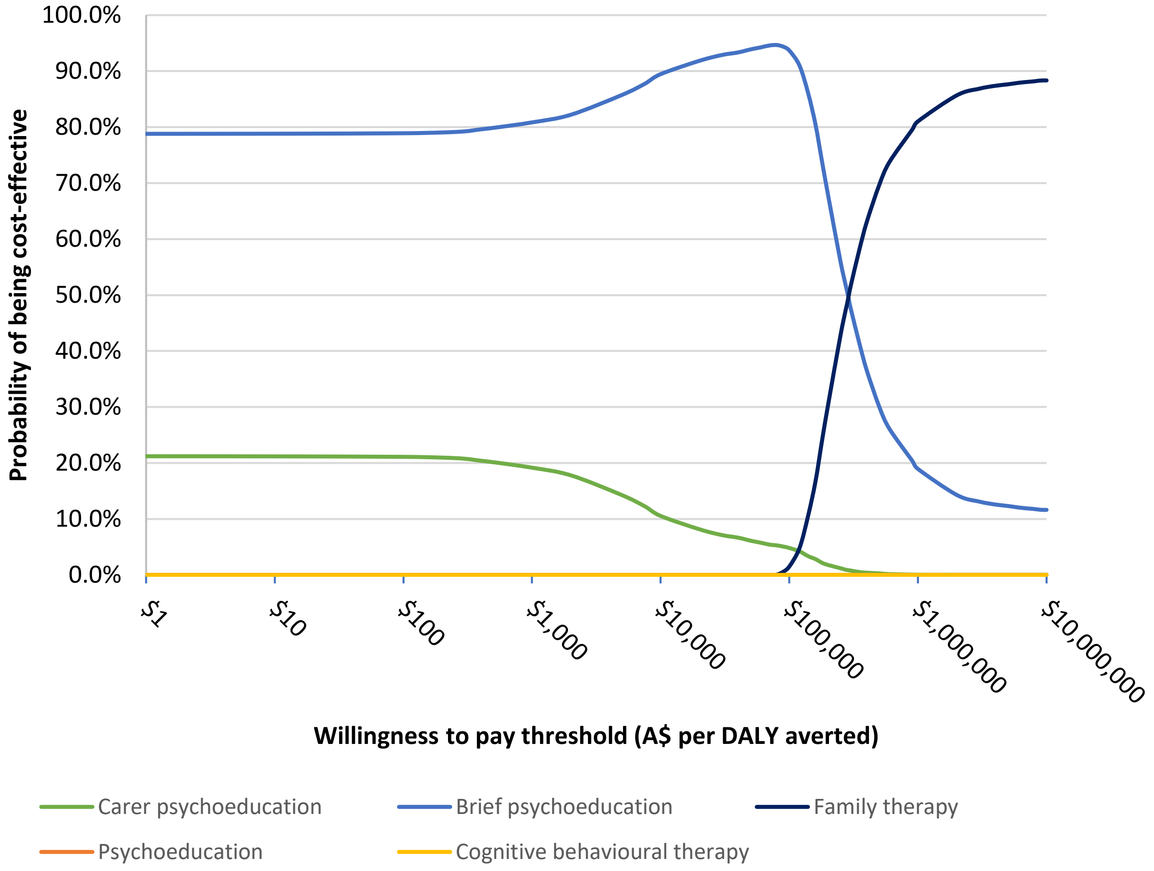

Figure S7. Cost-effectiveness acceptability frontier for the sensitivity analyses applying the effect to the mania phase using quality-adjusted life-years (QALYs) gained as the outcome measure

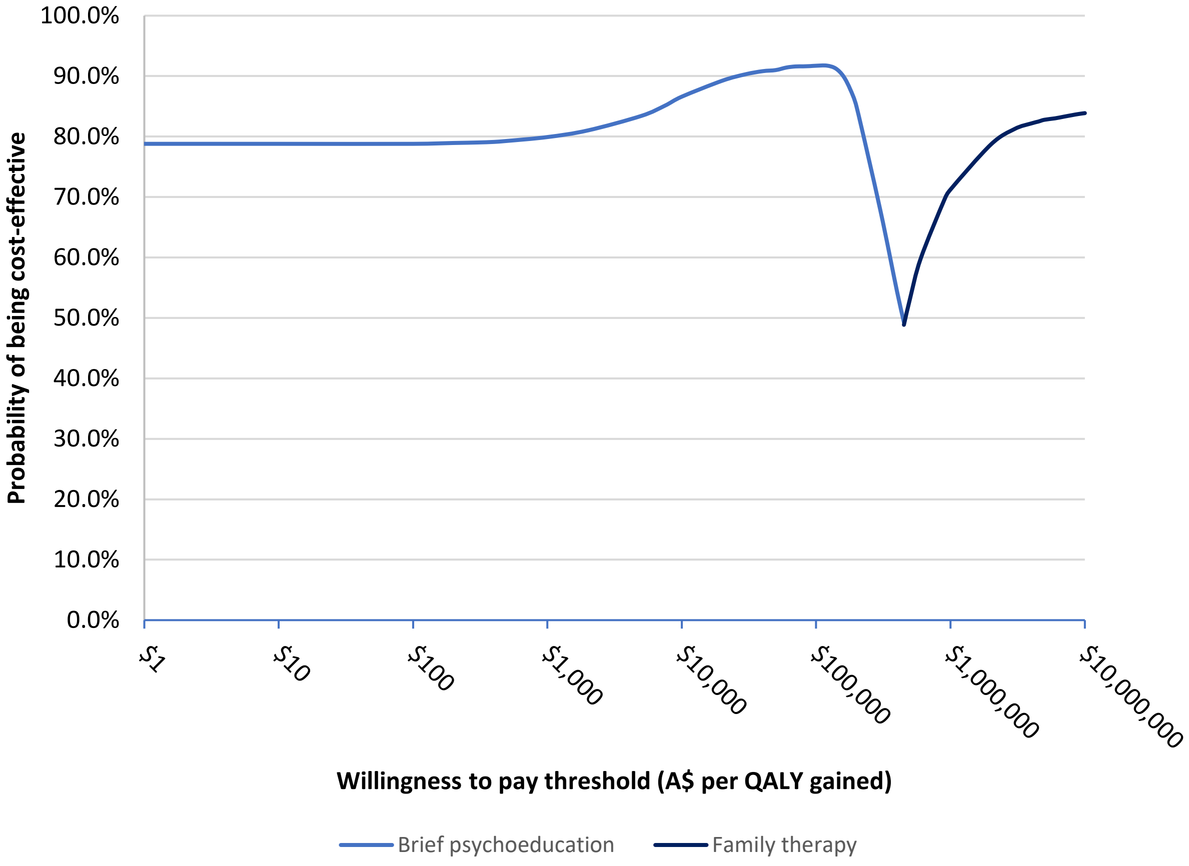

Figure S8. Cost-effectiveness acceptability frontier for the sensitivity analyses applying the effect to the mania phase using disability adjusted life year (DALYs) averted as the outcome measure

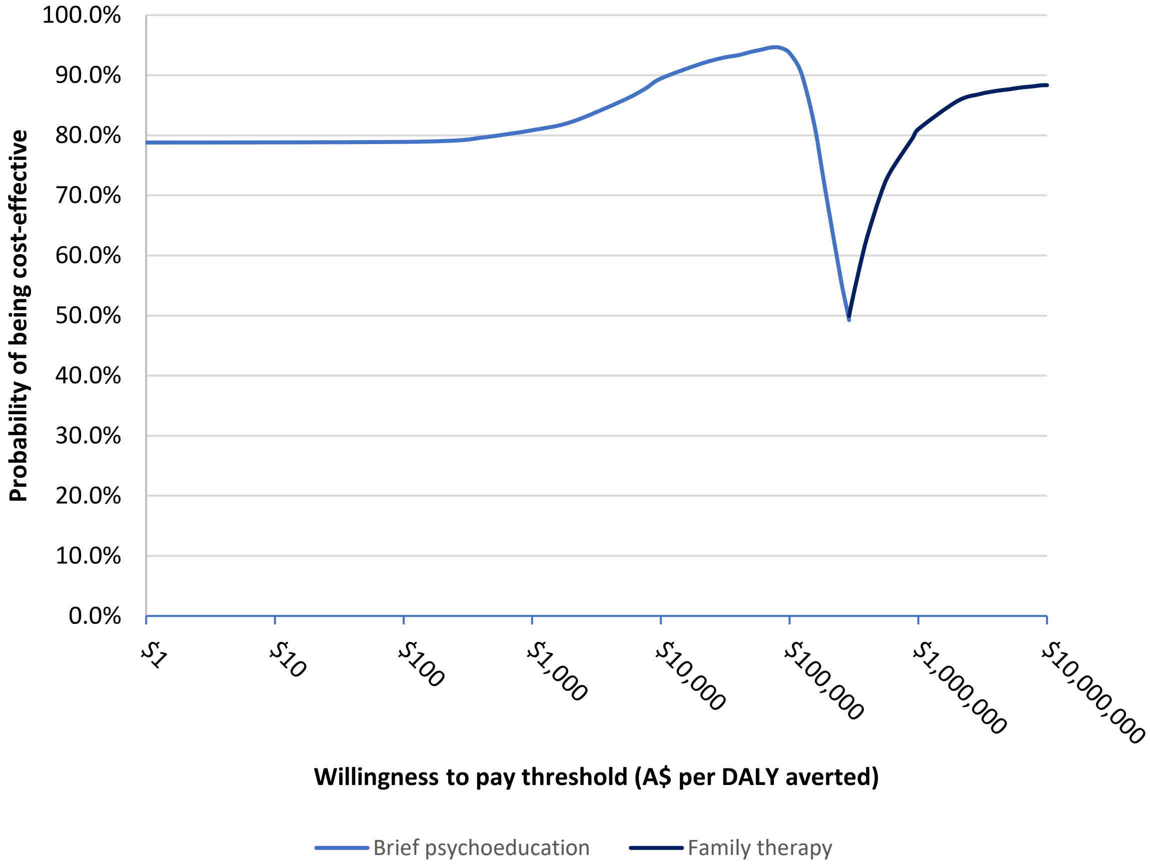

Figure S9. Cost-effectiveness plane for sensitivity analyses where the interventions are delivered by a psychologist using quality-adjusted life-years (QALYs) gained as the outcome measure

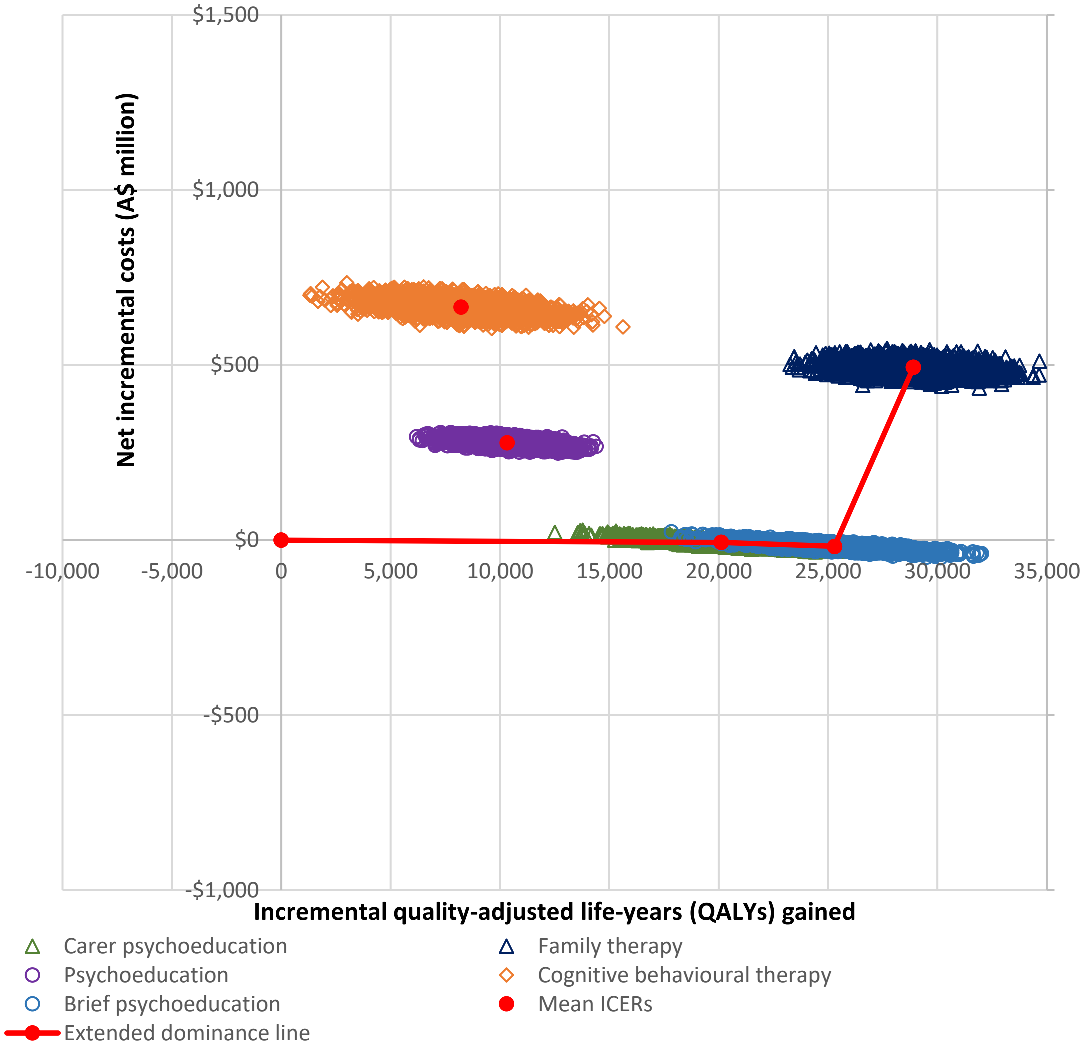

Figure S10. Cost-effectiveness plane for sensitivity analyses where the interventions are delivered by a psychologist using disability-adjusted life-years (DALYs) averted as the outcome measure

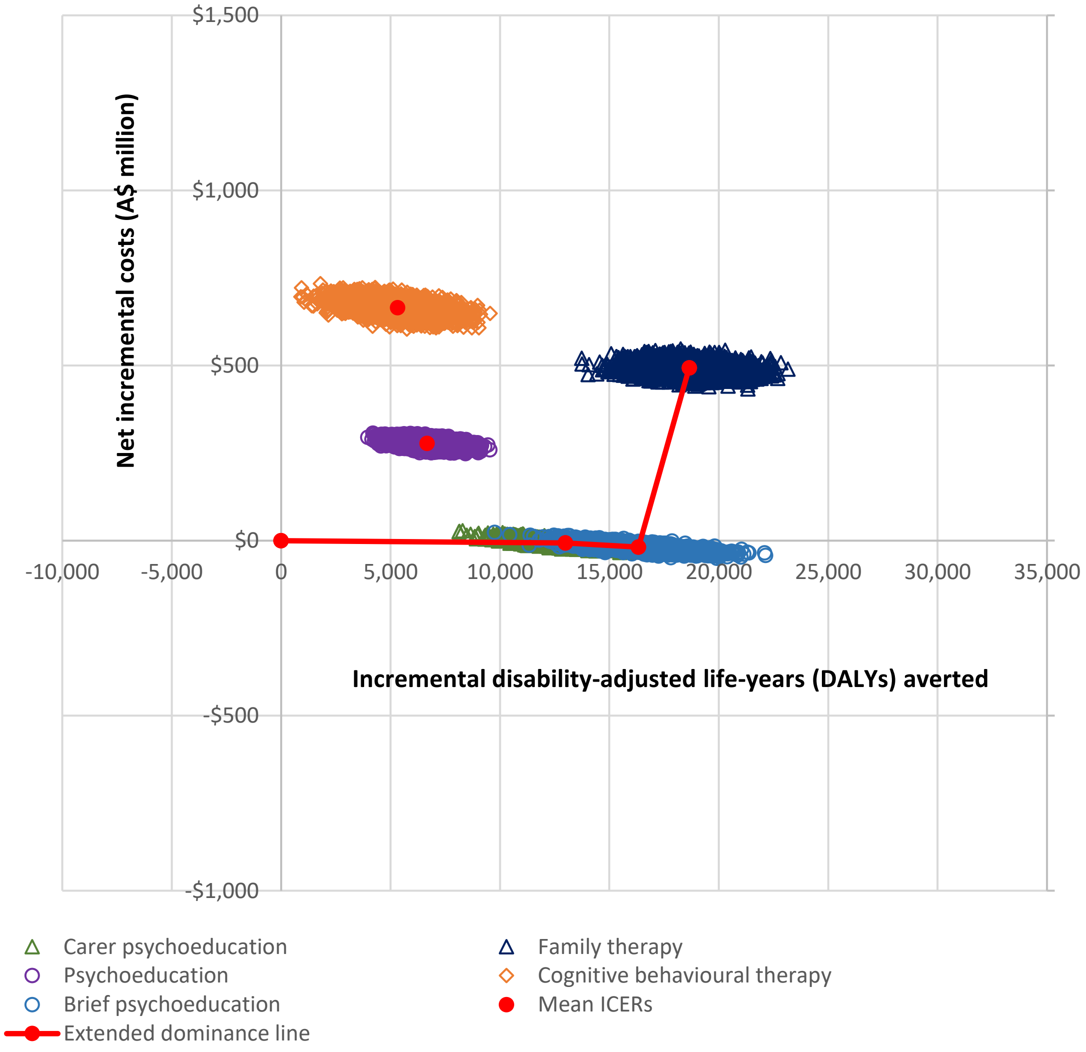

Figure S11. Cost-effectiveness acceptability curves for the sensitivity analyses where the interventions are delivered by a psychologist using quality-adjusted life-years (QALYs) gained as the outcome measure

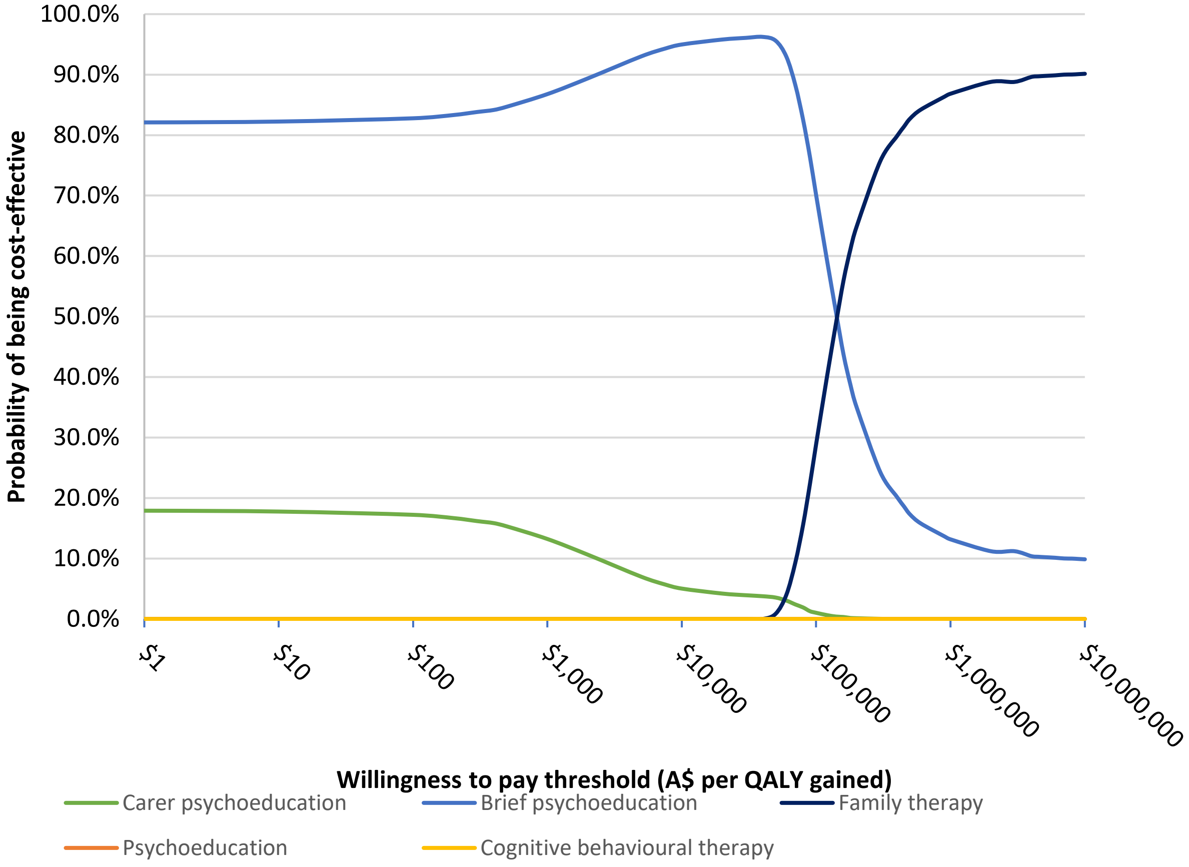

Figure S12. Cost-effectiveness acceptability curves for the sensitivity analyses where the interventions are delivered by a psychologist using disability-adjusted life-years (DALYs) averted as the outcome measure

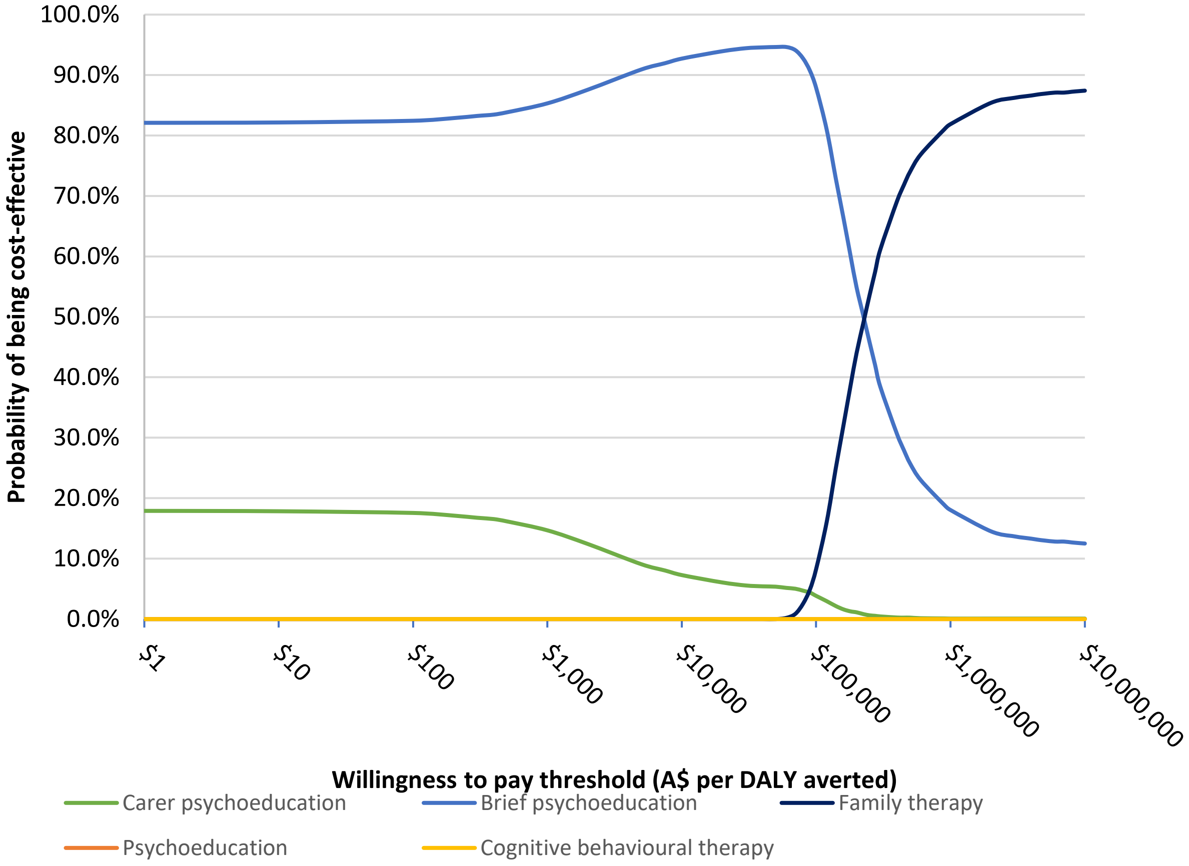

Figure S13. Cost-effectiveness acceptability frontier for the sensitivity analyses where the interventions are delivered by a psychologist using quality-adjusted life-years (QALYs) gained as the outcome measure

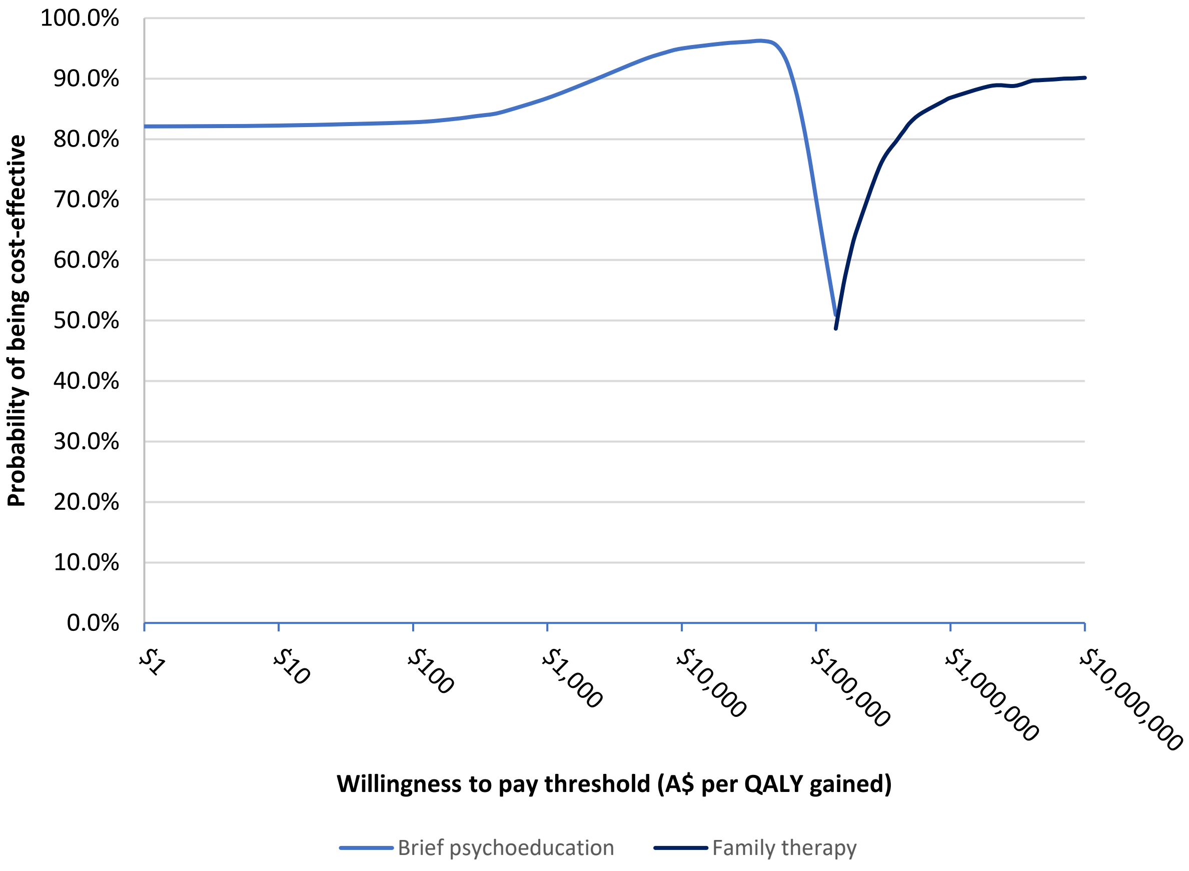

Figure S14. Cost-effectiveness acceptability frontier for the sensitivity analyses where the interventions are delivered by a psychologist using disability-adjusted life-years (DALYs) averted as the outcome measure

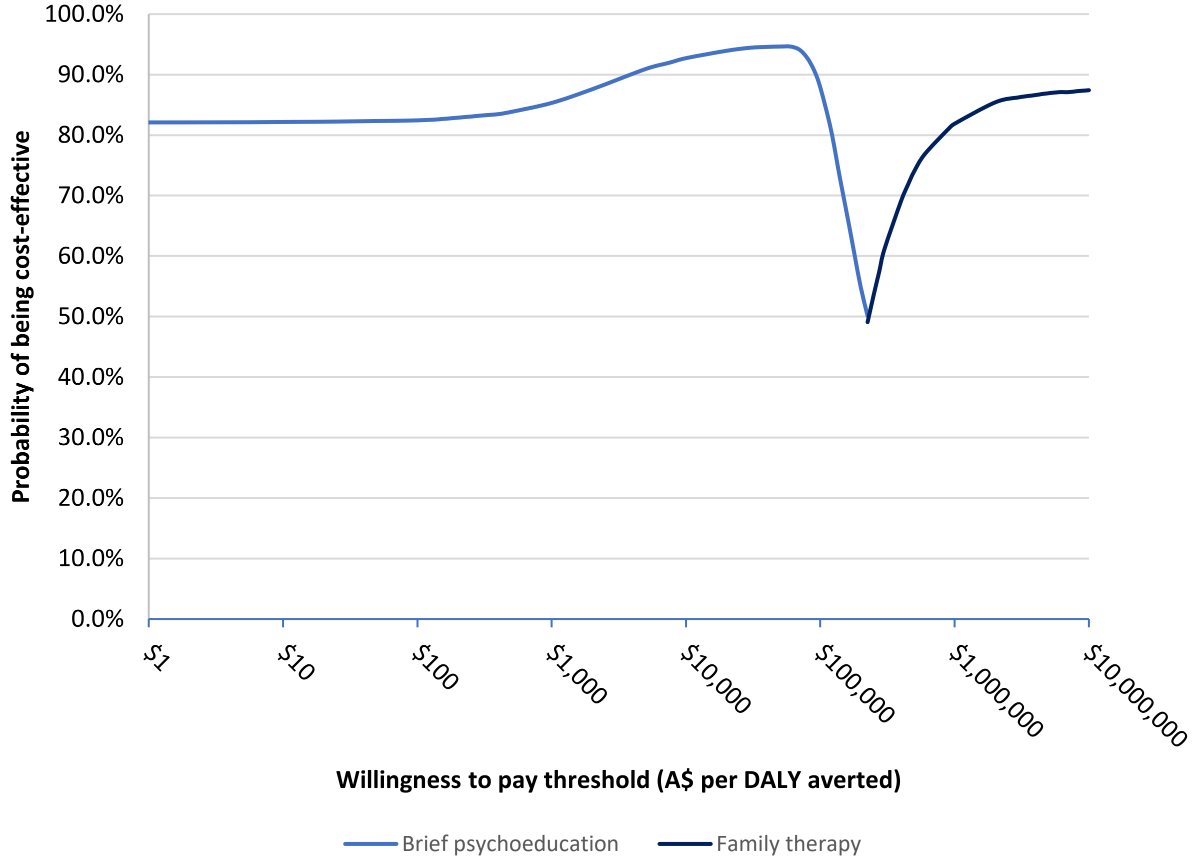

References

1. Cardoso Tde A, Campos Mondin T, Reyes AN, Zeni CP, Souza LD, da Silva RA, et al. Biological Rhythm and Bipolar Disorder: Twelve-Month Follow-Up of a Randomized Clinical Trial. J Nerv Ment Dis. 2015;203(10):792-7.
2. Colom F, Vieta E, Sanchez-Moreno J, Goikolea JM, Popova E, Bonnin CM, et al. J Affect Disord. 2009;112(1–3):30.
3. Harvey AG, Soehner AM, Kaplan KA, Hein K, Lee J, Kanady J, et al. Treating insomnia improves mood state, sleep, and functioning in bipolar disorder: a pilot randomized controlled trial. J Consult Clin Psychol. 2015;83(3):564-77.
4. Lobban F, Taylor L, Chandler C, Tyler E, Kinderman P, Kolamunnage-Dona R, et al. Enhanced relapse prevention for bipolar disorder by community mental health teams: cluster feasibility randomised trial. Br J Psychiatry. 2010;196(1):59-63.
5. Morriss R, Lobban F, Riste L, Davies L, Holland F, Long R, et al. Clinical effectiveness and acceptability of structured group psychoeducation versus optimised unstructured peer support for patients with remitted bipolar disorder (PARADES): a pragmatic, multicentre, observer-blind, randomised controlled superiority trial. Lancet Psychiatry. 2016;3(11):1029-38.
6. Parikh SV, Zaretsky A, Beaulieu S, Yatham LN, Young LT, Patelis-Siotis I, et al. A randomized controlled trial of psychoeducation or cognitive-behavioral therapy in bipolar disorder: a Canadian Network for Mood and Anxiety treatments (CANMAT) study [CME]. J Clin Psychiatry. 2012;73(6):803-10.
7. Rea MM, Tompson MC, Miklowitz DJ, Goldstein MJ, Hwang S, Mintz J. Family-focused treatment versus individual treatment for bipolar disorder: results of a randomized clinical trial. J Consult Clin Psychol. 2003;71(3):482-92.
8. Reinares M, Colom F, Sánchez-Moreno J, Torrent C, Martínez-Arán A, Comes M, et al. Impact of caregiver group psychoeducation on the course and outcome of bipolar patients in remission: a randomized controlled trial. Bipolar Disord. 2008;10(4):511-9.
9. Miklowitz DJ, George EL, Richards JA, Simoneau TL, Suddath RL. A randomized study of family-focused psychoeducation and pharmacotherapy in the outpatient management of bipolar disorder. Arch Gen Psychiatry. 2003;60(9):904-12.
10. Miklowitz DJ, Axelson DA, Birmaher B, George EL, Taylor DO, Schneck CD, et al. Family-focused treatment for adolescents with bipolar disorder: results of a 2-year randomized trial. Arch Gen Psychiatry. 2008;65(9):1053-61.
11. Miklowitz DJ, Schneck CD, George EL, Taylor DO, Sugar CA, Birmaher B, et al. Pharmacotherapy and family-focused treatment for adolescents with bipolar I and II disorders: a 2-year randomized trial. Am J Psychiatry. 2014;171(6):658-67.
12. Fayyazi Bordbar MR, Soltanifar A, Talaei A. Short-Term Family-Focused Psycho-Educational Program for Bipolar Mood Disorder in Mashhad. Iranian Journal of Medical Sciences. 2009;34.
13. Ball JR, Mitchell PB, Corry JC, Skillecorn A, Smith M, Malhi GS. A randomized controlled trial of cognitive therapy for bipolar disorder: focus on long-term change. J Clin Psychiatry. 2006;67(2):277-86.
14. Cochran SD. Preventing medical noncompliance in the outpatient treatment of bipolar affective disorders. J Consult Clin Psychol. 1984;52(5):873-8.
15. Gomes BC, Abreu LN, Brietzke E, Caetano SC, Kleinman A, Nery FG, et al. A randomized controlled trial of cognitive behavioral group therapy for bipolar disorder. Psychother Psychosom. 2011;80(3):144-50.
16. Jones SH, Smith G, Mulligan LD, Lobban F, Law H, Dunn G, et al. Recovery-focused cognitive-behavioural therapy for recent-onset bipolar disorder: randomised controlled pilot trial. Br J Psychiatry. 2015;206(1):58-66.

17. Lam D, Bright J, Jones S, Hayward P, Schuck N, Chisholm D, et al. Cognitive Therapy for Bipolar Illness—A Pilot Study of Relapse Prevention. Cognit Ther Res. 2000;24(5):503-20.
18. Lam DH, Hayward P, Watkins ER, Wright K, Sham P. Relapse prevention in patients with bipolar disorder: cognitive therapy outcome after 2 years. Am J Psychiatry. 2005;162(2):324-9.
19. Meyer TD, Hautzinger M. Cognitive behaviour therapy and supportive therapy for bipolar disorders: relapse rates for treatment period and 2-year follow-up. Psychol Med. 2012;42(7):1429-39.
20. Scott J, Paykel E, Morriss R, Bentall R, Kinderman P, Johnson T, et al. Cognitive-behavioural therapy for severe and recurrent bipolar disorders: randomised controlled trial. Br J Psychiatry. 2006;188:313-20.
21. D'Souza R, Piskulic D, Sundram S. A brief dyadic group based psychoeducation program improves relapse rates in recently remitted bipolar disorder: a pilot randomised controlled trial. J Affect Disord. 2010;120(1-3):272-6.
22. Nagy N, Sabry W, Khalifa D, Hashem R, Zahran N, Khalil A. Relapse rate and outcome correlates in Egyptian patients with bipolar disorder treated with behavioural family psychoeducation. Middle East Current Psychiatry. 2015;22:121-31.
23. Australian government Services Australia. Medicare Item Reports 2023 [Available from: http://medicarestatistics.humanservices.gov.au/statistics/mbs_item.jsp.
24. Australian Government Department of Health. Medicare Benefits Schedule Book Operating from 16 July 2021. 2021.
25. Australian Institute of Health and Welfare. Expenditure on mental health-related services; Data tables: Expenditure on mental health-related services tables 2020-21 2023 [Available from: https://www.aihw.gov.au/mental-health/topic-areas/expenditure.
26. Australian Institute of Health and Welfare. Community mental health care services; Data tables: Community mental health care services tables 2020-21 2022 [Available from: https://www.aihw.gov.au/mental-health/topic-areas/community-services#data.
27. IHACPA. National Hospital Cost Data Collection 2023 [Available from: https://www.ihacpa.gov.au/health-care/costing/national-hospital-cost-data-collection.
28. Australian Bureau of Statistics. Consumer Price Index, Australia 2023 [Available from: https://www.abs.gov.au/statistics/economy/price-indexes-and-inflation/consumer-price-index-australia/latest-release.
29. Andrews G, Tolkien II Team. Tolkien II: A Needs-based, Costed, Stepped-care Model for Mental Health Services Sydney: World Health Organization, Collaborating Centre for Classification in Mental Health; 2006.
